# Natalenamides A–C, Cyclic Tripeptides from the Termite-Associated *Actinomadura* sp. RB99

**DOI:** 10.3390/molecules23113003

**Published:** 2018-11-16

**Authors:** Seoung Rak Lee, Dahae Lee, Jae Sik Yu, René Benndorf, Sullim Lee, Dong-Soo Lee, Jungmoo Huh, Z. Wilhelm de Beer, Yong Ho Kim, Christine Beemelmanns, Ki Sung Kang, Ki Hyun Kim

**Affiliations:** 1 School of Pharmacy, Sungkyunkwan University, Suwon 16419, Korea; davidseoungrak@gmail.com (S.R.L.); pjsldh@naver.com (D.L.); jsyu@bu.edu (J.S.Y.); 2 Leibniz Institute for Natural Product Research and Infection Biology—Hans-Knöll-Institute, Beutenbergstraße 11a, 07745 Jena, Germany; rene.benndorf@hki-jena.de (R.B.); Christine.beemelmanns@hki-jena.de (C.B.); 3 College of Bio-Nano Technology, Gachon University, Seongnam 13120, Korea; sullimlee@gachon.ac.kr; 4 College of Korean Medicine, Gachon University, Seongnam 13120, Korea; vet4animal@hotmail.com (D.-S.L.); kkang@gachon.ac.kr (K.S.K.); 5 College of Pharmacy and Research Institute of Pharmaceutical Sciences, Seoul National University, Gwanak-gu, Seoul 08826, Korea; goodhjm112@snu.ac.kr; 6 Forestry and Agriculture Biotechnology Institute, University of Pretoria, Pretoria 0028, South Africa; wilhelm.debeer@fabi.up.ac.za; 7 SKKU—Advanced Institute of Nanotechnology (SAINT), Sungkyunkwan University, Suwon 16419, Korea; yhkim94@skku.edu

**Keywords:** fungus-growing termite, *Actinomadura* sp., tripeptides, natalenamides A–C, skin-whitening effects

## Abstract

In recent years, investigations into the biochemistry of insect-associated bacteria have increased. When combined with analytical dereplication processes, these studies provide a powerful strategy to identify structurally and/or biologically novel compounds. Non-ribosomally synthesized cyclic peptides have a broad bioactivity spectrum with high medicinal potential. Here, we report the discovery of three new cyclic tripeptides: natalenamides A–C (compounds **1**–**3**). These compounds were identified from the culture broth of the fungus-growing termite-associated *Actinomadura* sp. RB99 using a liquid chromatography (LC)/ultraviolet (UV)/mass spectrometry (MS)-based dereplication method. Chemical structures of the new compounds (**1**–**3**) were established by analysis of comprehensive spectroscopic methods, including one-dimensional (^1^H and ^13^C) and two-dimensional (^1^H-^1^H-COSY, HSQC, HMBC) nuclear magnetic resonance spectroscopy (NMR), together with high-resolution electrospray ionization mass spectrometry (HR-ESIMS) data. The absolute configurations of the new compounds were elucidated using Marfey’s analysis. Through several bioactivity tests for the tripeptides, we found that compound **3** exhibited significant inhibitory effects on 3-isobutyl-1-methylxanthine (IBMX)-induced melanin production. The effect of compound **3** was similar to that of kojic acid, a compound extensively used as a cosmetic material with a skin-whitening effect.

## 1. Introduction

The chemical analysis of protective bacterial symbionts of farming insects has attracted much attention among natural product chemists in recent years [1,2,3,4,5]. Using state-of-the-art nuclear magnetic resonance (NMR)/mass spectrometry (MS)-based dereplication processes and ecologically relevant bioassays, an impressive amount of structurally intriguing natural products, including several non-ribosomally synthesized cyclic peptides, have been uncovered from microbial symbionts [6]. The most prominent examples include the antifungal dentigerumycin, a cyclic depsipeptide isolated from a fungus-growing-ant-associated *Pseudonocardia* sp. [7], and the gerumycins A–C, piperazic acid-bearing cyclic depsipeptides identified from *Pseudonocardia* sp. [8].

Cyclic peptides have been shown to exhibit a broad range of biological activities, fostering several synthetic approaches toward these pharmacologically promising structures [9,10,11,12]. Over 40 cyclic peptide drugs are currently marketed and widely utilized in clinical environments, and approximately one new cyclic peptide drug enters the market every year, on average [13].

**Figure 1:**
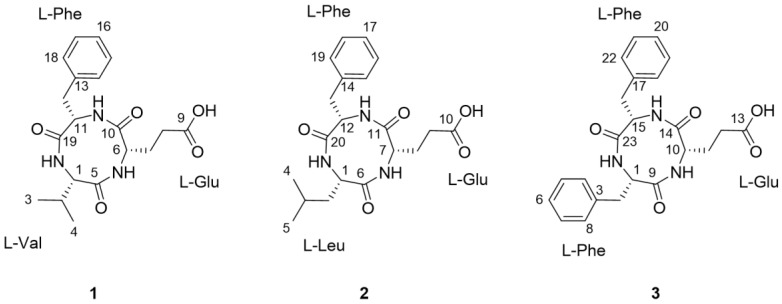
The chemical structures of natalenamides A–C (compounds **1**–**3**).

As part of our continuing endeavor to identify structurally novel and/or bioactive secondary metabolites from termite-associated microbes [14,15,16,17], we focused on the termite-associated *Actinomadura* sp. RB99, isolated from the fungus-growing termite, *Macrotermes natalensis*. Our liquid chromatography (LC)/ultraviolet (UV)/mass spectrometry (MS)-based dereplication strategy yielded potentially novel bioactive natural products. In this study, we report the isolation and chemical identification of three new cyclic tripeptides, natalenamide A–C (compounds **1**–**3**, Figure 1), using a LC/UV/MS-based dereplication method as well as their biological evaluations for cytotoxic, anti-inflammatory, and skin-whitening activities.

## 2. Results and Discussion

### 2.1. Liquid chromatography (LC)/Ultraviolet (UV)/Mass Spectrometry (MS)-Based Isolation of Compounds ***1***–***3***

*Actinomadura* sp. RB99 was isolated from the surface of a termite worker (*M. natalensis*). Phylogenetic analysis of a nearly complete 16S ribosomal ribonucleic acid (rRNA) sequence suggests that strain RB99 belongs to the genus *Actinomadura*, with the closest neighbor *Actinomadura nitritigenes* NBRC 15918^t^. Subsequent LC/UV/MS-based analysis of enriched culture extracts revealed a set of characteristic UV absorptions with unique molecular ion peaks containing nitrogen atoms. To identify the respective metabolites and investigate their activity, large-scale fermentation using optimized growth conditions (100 agar plates from 2 L ISP2 agar, pH 6, 10 days at 30 °C) and an established work-up and pre-purification procedure were applied [17]. Subsequent LC-UV/MS-guided fractionation and repetitive semi-preparative high performance liquid chromatography (HPLC) resulted in isolation of three metabolites with a unique NMR/MS pattern. We performed growth and media studies using different salt (NaCl, KBr 1–3%) and media compositions (ISP1-6 media) to mimic the natural environment and stimulate metabolite production, which also led to the presence of the three new metabolites (see Supplementary Figure S19).

### 2.2. Structural Elucidation of the Compounds

**Figure 2:**
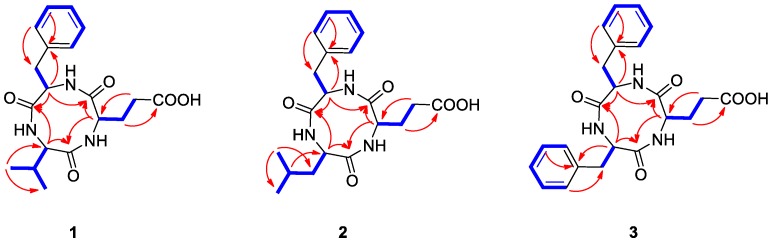
Key COSY (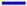) and HMBC (→) correlations for compounds **1**–**3**.

Natalenamide A (**1**) was acquired as an amorphous powder. The molecular formula of **1** was determined to be C_19_H_25_N_3_O_5_ from the sodium-adducted molecular ion at *m*/*z* 398.1691 [M + Na]^+^ (calculated for C_19_H_25_N_3_O_5_Na, 398.1692) using positive-ion mode high-resolution electrospray ionization mass spectrometry (HR-ESIMS) data. The infrared (IR) spectrum showed a strong absorption band at 1670 cm^−1^, suggesting the presence of amide groups in the molecule. The detailed analysis of ^1^H NMR data of **1** (Table 1) indicated the typical signals of a peptide skeleton, displaying two methyl signals (*δ*_H_ 0.91 (3H, d, *J* = 7.0 Hz, H-3) and 0.92 (3H, d, *J* = 7.0 Hz, H-4)), three methylene signals (*δ*_H_ 1.95 (1H, m, H-7a), 2.27 (1H, m, H-8a), 2.36 (1H, m, H-8b), 2.48 (1H, m, H-7b), 2.97 (1H, dd, *J* = 14.0, 8.0 Hz, H-12a), and 3.20 (1H, dd, *J* = 14.0, 5.0 Hz, H-12b)), four methine signals (*δ*_H_ 2.02 (1H, m, H-2), 4.16 (1H, d, *J* = 7.5 Hz, H-1), 4.20 (1H, dd, *J* = 8.5, 4.5 Hz, H-6), and 4.63 (1H, dd, *J* = 8.0, 5.0 Hz, H-11)), and five overlapped aromatic protons (*δ*_H_ 7.18 (1H, m, H-16), 7.23 (2H, m, H-14/18), and 7.24 (2H, m, H-15/17)). The ^13^C NMR spectrum (Table 1), with assistance from the HSQC and HMBC spectra, showed a total of 19 carbon resonances responsible for two methyl signals (*δ*_C_ 18.9 and 19.9), three methylene signals (*δ*_C_ 27.0, 30.6, and 38.8), nine methine signals (*δ*_C_ 32.1, 55.6, 58.0, 60.4, 127.8, 129.5 (×2), and 130.6 (×2)), including five aromatic carbons, one olefinic carbon signal (*δ*_C_ 138.8), and four carbonyl carbon signals (*δ*_C_ 173.2, 173.3, 174.8, and 181.3). Combined with the above spectroscopic data, detailed inspection of two-dimensional (2D) NMR (^1^H-^1^H COSY, HSQC, and HMBC experiments) revealed the presence of three amino acids in **1**: glutamate (Glu), phenylalanine (Phe), and valine (Val) (Figure 2). The connectivity of these amino acids was determined by the HMBC correlations of H-1/C-5 and C-19, H-11/C-10 and C-19, and H-6/C-5 and C-10 (Figure 2). To identify the absolute configuration of **1**, Marfey’s method was applied to determine the stereochemistry of α-H multiples (C-1, C-6, and C-11). The acid hydrolysates of **1** and standard amino acids (L/D-Glu, Phe, and Val) were derivatized with 1-fluoro-2,4-dinitrophenyl-5-L-alanineamide (L-FDAA). Successively, the derivatized mixture of **1** and the standard amino acids were analyzed by LC/MS to examine their retention time. The absolute configurations of the Glu, Phe, and Val moieties were all determined to be L-forms, based on the retention times of the L-FDAA derivatives of **1** compared with those of the standard amino acids. Thus, the structure of **1** was elucidated as the cyclic tripeptide shown in Figure 1.

Natalenamide B (**2**) was isolated as an amorphous powder. Its molecular formula (C_20_H_27_N_3_O_5_) was deduced from hydrogen and sodium adducted molecular ion peaks at 390.2032 [M + H]^+^ (calculated for C_20_H_28_N_3_O_5_, 390.2029) and 412.1849 [M + Na]^+^ (calculated for C_20_H_27_N_3_O_5_Na, 412.1848), respectively. Compared with the molecular formula and NMR spectral data of **1**, compound **2** appeared to have an additional CH_2_ group in the peptide skeleton. Comprehensive scrutiny of the one-dimensional (1D) (^1^H and ^13^C NMR) and 2D NMR (^1^H–^1^H COSY, TOCSY, HSQC, and HMBC) spectra of **2** revealed the existence of three amino acid residues: Glu, Phe, and Leu (leucine). The sequence of these amino acids was constructed based on the HMBC correlations of H-1/C-6 and C-20, H-12/C-11 and C-20, and H-7/C-6 and C-11 (Figure 2). To determine the absolute configuration of **2**, derivatization of the Glu, Phe, and Leu residues with L-FDAA was performed and then analyzed by LC/MS. In the LC/MS data, L-Glu, L-Phe, and L-Leu were absolutely detected by comparing the retention times for the L-FDAA derivatives of **2** with those of the standard amino acids. Thus, the chemical structure of **2** was established as the cyclic tripeptide shown in Figure 1.

The positive-mode HR-ESIMS data of natalenamide C (**3**) displayed hydrogen and sodium adducted molecular ions at 424.1870 [M + H]^+^ (calculated for C_23_H_26_N_3_O_5_, 424.1872) and 446.1694 [M + Na]^+^ (calculated for C_23_H_25_N_3_O_5_Na, 424.1692). This suggested that its molecular formula was C_23_H_25_N_3_O_5_. A detailed analysis of the 1D and 2D NMR spectra of **3** indicated that its chemical structure was similar to that of **2**, except Leu was substituted with Phe in **3**. The connectivity of the three identified amino acids in **3** was verified using the HMBC correlations of H-1/C-9 and C-23, H-15/C-14 and C-23, and H-10/C-9 and C-14 (Figure 2). By applying Marfey’s method to compound **3**, the absolute configurations of the α-H multiples (C-1, C-10, and C-15) were elucidated to be one L-Glu and two L-Phe moieties. Accordingly, the complete structure of **3** was determined as shown in Figure 1.

Natalenamides A–C are tricyclic peptides, presumably of non-ribosomal origin. Currently, several bioactive cyclic tripeptides have been characterized as natural products: cytotoxic 17-membered cyclic tripeptides (e.g., OF4949 I–IV) isolated from *Penicillium rugulosum* [18]; antifungal sclerotiotides A−K, identified from the fermentation broth of halotolerant bacteria [10]; and antiproliferative psychrophilin E, isolated from a mixed culture of two marine alga-derived fungal strains of the genus *Aspergillus* [19].

### 2.3. Biological Activities of the Compounds ***1***–***3***

**Figure 3:**
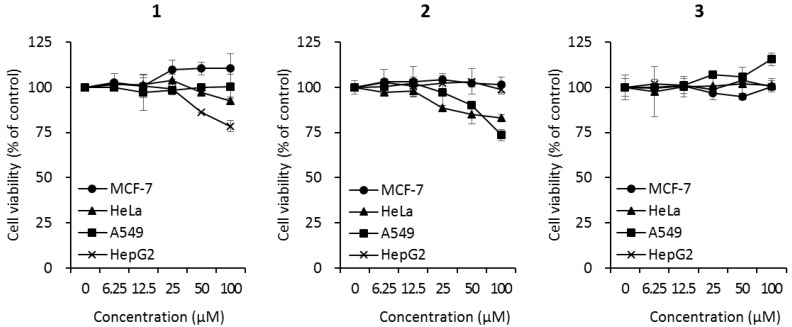
Cytotoxicity of compounds **1**–**3** against human cancer cell lines (MCF7 breast cancer cells, HeLa cervical cancer cells, A549 lung cancer cells, and HepG2 liver cancer cells). Cells were treated with compounds at the indicated concentrations. After 72 h of incubation, cell viability was analyzed according to the manufacturer’s instructions using the EZ-CyTox cell viability assay kit.

Since cyclic peptides have been reported to exhibit a wide range of biological activities, the biological effects of the isolated cyclic tripeptides (**1**–**3**) were evaluated using three bioactivity tests. The cytotoxicity of compounds **1**–**3** was investigated against four cancer cell lines (MCF7 breast cancer cells, HeLa cervical cancer cells, A549 human lung cancer cells, and HepG2 liver cancer cells) at different concentrations (0, 6.25, 12.5, 25, 50, and 100 μM). Compound **1** did not affect the cell viability of MCF-7, HeLa, or A549 cells. However, the treatment of HepG2 cells with 100 μM of compound **1** decreased cell viability to 78.5 ± 3.2% (Figure 3). Compound **2** reduced the cell viabilities of HeLa and A549 cells to 82.9 ± 2.1% and 73.5 ± 3.0%, respectively, but did not affect the viability of MCF-7 or HepG2 cells (Figure 3). Compound **3** did not affect the viability of any of the cell lines (Figure 3).

**Figure 4:**
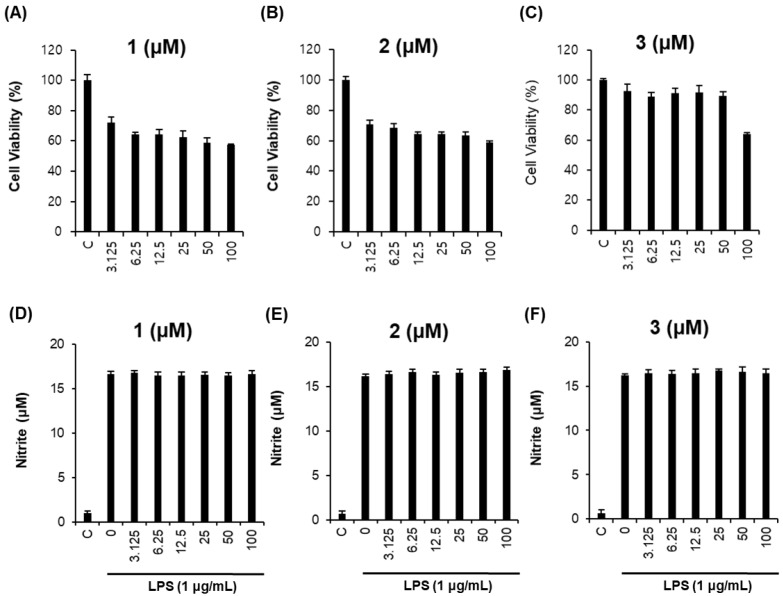
Inhibitory effects of compounds **1**–**3** on lipopolysaccharide (LPS)-induced NO production in RAW264.7 cells. (A–C) Effects of compounds **1**–**3** on cell viability. (D–F) Inhibitory effects of compounds **1–3** on LPS-induced NO production in RAW264.7 cells.

Next, RAW264.7 macrophages were used to investigate the anti-inflammatory effects of the isolated compounds. To eliminate error in the production of NO caused by changing cell survival rates, non-toxic concentrations of each compound were examined. As shown in Figure 4, compounds **1**–**3** had few cytotoxic effects in RAW264.7 cells (Figure 4A–C) and exerted no inhibitory effects on NO production in lipopolysaccharide (LPS)-stimulated RAW264.7 cells (Figure 4D–F).

**Figure 5:**
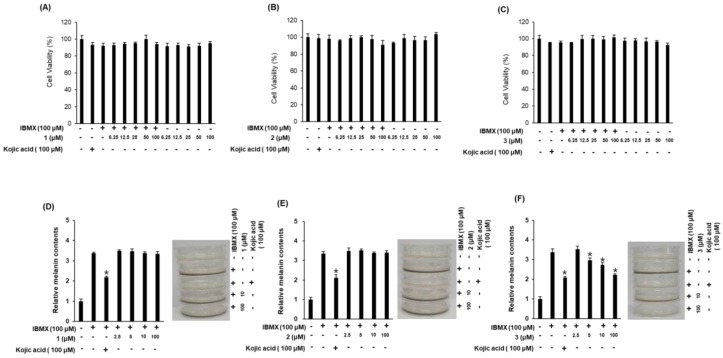
Inhibitory effects of compounds **1**–**3** on melanin content in B16F10 cells. (**A**–**C**) Effects of compounds **1**–**3** on cell viability. (**D**–**F**) Inhibitory effects of compounds **1**–**3** on IBMX-induced melanin production in B16F10 melanoma cells (mean ± SD, * *p* < 0.05 compared to the IBMX-treated value).

The inhibitory effects of compounds **1**–**3** on the melanin content in B16F10 cells were examined as evidence of their skin-whitening activities (Figure 5). Since changes in cell viability cause errors in the production and measurement of melanin, we first examined the effects of compounds **1**–**3** on B16F10 cell survival. Compounds **1**–**3** exerted no cytotoxic effects in B16F10 cells at any of the concentrations used (Figure 5A–C). We then assessed the inhibitory effects of the compounds on 3-isobutyl-1-methylxanthine (IBMX)-induced melanin production in B16F10 cells (Figure 5D–F). IBMX, a well-known stimulator of melanogenesis, induces a significant increase in melanin production following a single treatment in melanoma cells. Among the compounds evaluated, compound **3** (at 5–100 μM) exhibited significant inhibitory effects on IBMX-mediated melanin synthesis in a dose-dependent manner. Kojic acid, our positive control, has been extensively used as a cosmetic material with skin-whitening effects [20]. The inhibitory effect of compound **3** on IBMX-induced melanin production was similar to that of kojic acid (Figure 5F), suggesting that compound **3** functions as a potent inhibitor of IBMX-induced melanin production in B16F10 melanoma cells. Furthermore, compound **3** was contaminated with a small amount of impurities, which were determined to be fatty acid analogues by the interpretation of NMR spectroscopic data and LC/MS analysis (Supplementary Materials, Figures S11–S15 and S23). To identify the activity of the impurities, additional experiments with the fatty acid analogues were conducted for their inhibitory effects on IBMX-induced melanin production in B16F10 cells, which revealed that the tested compounds had no whitening effect (Supplementary Materials, Figure S24). Thus, although we cannot absolutely exclude that the impurities in compound **3** are responsible for the inhibitory effect on IBMX-induced melanin production, this seems unlikely.

## 3. Materials and Methods

### 3.1. General Experimental Procedures

Infrared spectra were acquired on a Bruker IFS-66/S FT-IR (Bruker, Billerica, MA, USA) spectrometer. The electrospray ionization and HR-ESIMS spectra were measured on a SI-2/LCQ DecaXP liquid chromatography (LC)-mass spectrometer (Thermo Fisher Scientific, Waltham, MA, USA). NMR spectra, including ^1^H–^1^H COSY, HSQC, and HMBC experiments, were performed using a Varian UNITY INOVA 800 NMR spectrometer (Varian, Palo Alto, CA, USA) operating at 800 MHz (^1^H) and 200 MHz (^13^C), with chemical shifts given in ppm (δ). Preparative HPLC utilized a Waters 1525 Binary HPLC pump with Waters 996 Photodiode Array Detector (Waters Corporation, Milford, CT, USA). Silica gel 60 (230–400 mesh, Merck, Kenilworth, NJ, USA) and RP-C18 silica gel (230–400 mesh, Merck, were used for column chromatography. Semi-preparative HPLC used a Shimadzu Prominence HPLC System with SPD-20A/20AV Series Prominence HPLC ultraviolet–visible (UV–Vis) Detectors (Shimadzu, Tokyo, Japan). LC/MS analyses were carried out on an Agilent 1200 Series HPLC system (Agilent Technologies, Santa Clara, CA, USA) equipped with a diode array detector and a 6130 Series ESI mass spectrometer with an analytical Kinetex (4.6 × 100 mm, 3.5 μm). Merck pre-coated silica gel F254 plates and RP-18 F254s plates were used for thin-layer chromatography (TLC). Spots were detected on TLC under UV light or by heating after spraying with anisaldehyde-sulfuric acid solution.

### 3.2. Microbial Material

*Actinomadura* sp. RB99 was isolated from the surface of a termite worker of the genus, *M. natalensis*, (colony Mn103, GPS S25 43 45.9 E28 14 08.9) in January 2010. Biomaterial was placed into clean plastic bags and processed within one day of collection. Termites were washed in sterile deionized water, and bacteria were isolated by plating the resulting suspensions on low-nutrient media with chitin (per liter: 4 g chitin, 0.7 g K_2_HPO_4_, 0.3 g KH_2_PO_4_, 0.5 g MgSO_4_∙5H_2_O, 0.01 g FeSO_4_∙7H_2_O, 0.001 g ZnSO_4_, 0.001 g MnCl_2_, and 20 g agar) [21]. Isolates with *Actinobacteria*-like morphology were transferred to ISP2 agar (per liter: 10 g malt extract, 4 g yeast extract, 4 g glucose, and 20 g agar), and sub-cultured until pure isolates were obtained.

### 3.3. DNA Extraction and Polymerase Chain Reaction Amplification

*Actinomadura* sp. RB99 was grown in nutrient-rich liquid media ISP2 for five to seven days at 30 °C. Cells were harvested, and genomic DNA was extracted using the GenJet genomic DNA purification kit (#K0721, Thermo Scientific, Waltham, MA, USA) following the manufacturer’s instructions with the following changes: (a) lysozyme treatment was extended to 40 min, and (b) proteinase K treatment was extended to 40 min. DNA was quantified photometrically using a Nanodrop Lite spectrometer (Thermo Scientific). For phylogenetic studies, the 16S rRNA gene was amplified using the primer set, 1492R/27F. Each amplification reaction was prepared in a 25 μL final reaction volume containing 7.25 μL of distilled water, 5 μL of HF buffer, 5 μL of each primer (2.5 μM), 0.5 μL of dNTPs (10 μM), 0.25 μL of Phusion High-Fidelity DNA polymerase (New England Biolabs, Ipswich, MA, USA), and 2 μL of extracted DNA (template). Polymerase chain reaction (PCR) was performed under the following conditions: 98 °C for 38 s; 32 cycles of 98 °Cfor 30 s, 52 °C for 45 s, 72 °C for 1 min 20 s; and a final extension of 72 °C for 8 min. The PCR product was visualized by agarose gel electrophoresis, and PCR reactions were purified using a PCR purification kit (Thermo Scientific). DNA fragments were sequenced at GATC (Konstanz, Germany).

### 3.4. Sequencing and Species Identification

Sequences were assessed for purity and mismatches using BioEdit [22]. The forward and reverse sequences obtained for each strain were assembled with BioEdit and tested for chimeras using DECIPHER (http://decipher.cee.wisc.edu/FindChimerasOutputs.html). Resulting sequences were deposited in GenBank (accession number: KY558684). Blast analyses with almost-complete 16S rRNA sequences (1368 bp) were performed using the National Center for Biotechnology Information (NCBI) database (reference RNA sequences). The results indicated that strain RB99 is a member of the genus, *Actinomadura*. Sequences of the first 10 hits were downloaded from the NCBI database and aligned with the 16S rRNA sequence of *Actinomadura* sp. RB99 using the Sina sequences alignment service [23]. Two different phylogenetic trees were reconstructed with neighbor-joining or maximum-likelihood algorithms using MEGA software version 7.0.26 [24,25,26]. The evolutionary distance model of Tamura and Nei was used to generate evolutionary distance matrices for the maximum-likelihood and neighbor-joining algorithms, with deletion of complete gaps and missing data [27]. For the maximum-likelihood algorithm, discrete gamma distribution was used (+G), and the rate variation model allowed for some sites to be evolutionarily invariable (+I). For the neighbor-joining algorithm, the rate variation among sites was modeled with a gamma distribution (+G). The confidence values of nodes were evaluated by bootstrap analyses based on 1000 resampling steps [28].

### 3.5. Extraction and Isolation

*Actinomadura* sp. RB99 was grown in 50 mL ISP2 broth for seven days at 30 °C (pre-culture) and used to inoculate 100 ISP2 agar plates. Plates were incubated for 10 days at 30 °C, cut into small pieces, consolidated, and immersed overnight in MeOH. The MeOH phase was filtered and evaporated under reduced pressure. The MeOH extract (20 g) was dissolved in distilled water (700 mL) and then solvent-partitioned with EtOAc (700 mL) three times, affording 1.1 g of residue. The EtOAc-soluble fraction (1.1 g) from the MeOH extract was loaded onto a silica gel (230–400 mesh) column for chromatography and eluted with a gradient solvent system of CH_2_Cl_2_-MeOH (100:1 to 1:1, *v*/*v*). This provided six fractions (A–F), which were subjected to LC-UV/MS-based analysis. Dereplication analysis using an in-house UV spectral library suggested the existence of minor peptide analogues in fraction E. These displayed a simple UV pattern at around 220 nm and unique molecular formula ion peaks containing nitrogen atoms. The polar fraction E (233 mg) was fractionated by preparative reversed-phase HPLC (Phenomenex Luna C18, 250 × 21.2 mm i.d., 5 µm, Torrance, CA, USA) using CH_3_CN/H_2_O (1:9 to 9:1, *v*/*v*, gradient system, flow rate: 5 mL/min) to yield five sub-fractions (E1–E5). Sub-fraction E2 (27 mg) was purified by a semi-preparative reversed-phase HPLC (Phenomenex Luna C18, 250 × 10.0 mm i.d., 5 μm) with 33% MeOH/H_2_O (isocratic system, flow rate: 2 mL/min) to yield compound **1** (5.8 mg, *t*_R_ = 57.0 min). Compounds **2** (1.6 mg, *t*_R_ = 42.0 min) and **3** (1.5 mg, *t*_R_ = 55.0 min) were isolated from sub-fraction E3 (15 mg) by semi-preparative reverse-phase HPLC eluting 43% MeOH/H_2_O (isocratic system, flow rate: 2 mL/min).

#### 3.5.1. Natalenamide A (**1**)

Amorphous powder. ${\left[\alpha \right]}_{D}^{25}$ -16.4 (*c* 0.02, MeOH); IR (KBr) ν_max_ 3370, 1723, 1670, 1409, 1054 cm^−1^; UV (MeOH) λ_max_ (log *ε*) 208 (3.90) nm; ^1^H (800 MHz) and ^13^C NMR (200 MHz) data, see Table 1; HR-ESIMS (positive ion-mode) *m*/*z* 398.1691 [M + Na]^+^ (calculated for C_19_H_25_N_3_O_5_Na, 398.1692).

#### 3.5.2. Natalenamide B (**2**)

Amorphous powder. ${\left[\alpha \right]}_{D}^{25}$ -18.4 (*c* 0.05, MeOH); IR (KBr) ν_max_ 3352, 1756, 1630, 1392, 1031 cm^−1^; UV (MeOH) λ_max_ (log *ε*) 210 (3.78) nm; ^1^H (800 MHz) and ^13^C NMR (200 MHz) data, see Table 1; HR-ESIMS (positive ion-mode) 390.2032 [M + H]^+^ (calculated for C_20_H_28_N_3_O_5_, 390.2029) and 412.1849 [M + Na]^+^ (calculated for C_20_H_27_N_3_O_5_Na, 412.1848).

#### 3.5.3. Natalenamide C (**3**)

Amorphous powder. ${\left[\alpha \right]}_{D}^{25}$ -10.2 (*c* 0.02, MeOH); IR (KBr) ν_max_ 3225, 1746, 1685, 1359, 1027 cm^−1^; UV (MeOH) λ_max_ (log *ε*) 209 (3.90) nm; ^1^H (800 MHz) and ^13^C NMR (200 MHz) data, see Table 1; HR-ESIMS (positive ion-mode) 424.1870 [M + H]^+^ (calculated for C_23_H_26_N_3_O_5_, 424.1872) and 446.1694 [M + Na]^+^ (calculated for C_23_H_25_N_3_O_5_Na, 424.1692).

### 3.6. Acid Hydrolysis of Compounds ***1***–***3***

An amount totaling 0.4 mg of each compound (**1**–**3**) was hydrolyzed with 6 N HCl (500 µL) for 1 h at 110 °C. After cooling to room temperature, the hydrolysates of **1**–**3** were evaporated to remove traces of HCl. Distilled water (500 µL) was added to the hydrolysate mixtures and then evaporated to remove traces of HCl; this process was performed three times.

### 3.7. Determination of the Absolute Configuration of Amino Acids in ***1***–***3***

The hydrolysate mixtures (**1**–**3**), as well as the standard amino acids (L/D-Leu, Glu, Phe, and Val), were dissolved in 1 N NaHCO_3_ (100 µL) and then treated with 50 µL of L-FDAA (10 mg/mL in acetone). Successively, each hydrolysate was heated for 10 min at 80 °C. Each mixture was quenched with 2 N HCl (50 µL) and concentrated in vacuo. The residue was dissolved in 300 µL of MeOH. Each aliquot (5 µL) acquired from the hydrolysate mixtures was directly injected onto the LC/MS (Phenomenex Luna C18, 4.6 × 100 mm, 3.5 μm, flow rate of0.3 mL/min), and a full scan in positive and negative ion modes (scan range from *m*/*z* 100 to 1000) was applied to identify the retention times of the L-FDAA-derivatized amino acids. The mobile phase, consisting of formic acid in distilled water (0.1% *v*/*v*) (A) and acetonitrile (B), was carried with a gradient solvent system as follows: 20–40% (B) for 10 min, 100% (B) isocratic for 5 min, and then 20% (B) isocratic for 5 min, to conduct a post-run washing procedure for the column. The retention times of the L-FDAA derivatized amino acids used as standards were 16.7 min (L-Glu, *m*/*z* 400 [M + H]^+^), 18.2 min (D-Glu, *m*/*z* 400 [M + H]^+^), 22.1 min (L-Val, *m*/*z* 370 [M + H]^+^), 25.1 min (D-Val, *m*/*z* 370 [M + H]^+^), 25.6 min (L-Phe, *m*/*z* 418 [M + H]^+^), 27.0 min (D-Phe, *m*/*z* 418 [M + H]^+^), 25.5 min (L-Leu, *m*/*z* 384 [M + H]^+^), and 28.2 min (D-Leu, *m*/*z* 384 [M + H]^+^). The retention times of the derivatized hydrolyzates of **1**–**3** were L-Glu (16.9 min), L-Phe (25.7 min), and L-Val (22.5 min) from **1**; L-Glu (16.7 min), L-Phe (25.7 min), and L-Leu (25.6 min) from **2**; and L-Glu (16.9 min) and L-Phe (25.7 min) from **3**.

### 3.8. Cytotoxic Assays Using Cancer Cells

MCF7, HeLa, and A549 cells were purchased from the American Type Culture Collection (Rockville, MD, USA). HepG2 cells were purchased from the Korean Cell Line Bank (Seoul, Korea). MCF7 cells were cultured in Roswell Park Memorial Institute (RPMI) 1640 medium supplemented with 10% fetal bovine serum. HeLa and A549 cells were cultured in Dulbecco’s minimal essential medium (DMEM, Cellgro, Manassas, VA, USA) supplemented with 10% fetal bovine serum. HepG2 cells were cultured in Minimum Essential Medium supplemented with 10% fetal bovine serum. Culture conditions were maintained at 37 °C in a humidified atmosphere containing 5% CO_2_. Cytotoxicity was tested on these four human cell lines (MCF7, HeLa, A549, and HepG2) using the EZ-CyTox cell viability assay kit (Dojindo Laboratories, Kumamoto, Japan). Briefly, 5 × 10^3^ cells /well were seeded in 96-well plates. After 24 h, cells were treated with compounds at the indicated concentrations. After 72 h of incubation, the EZ-CyTox cell viability assay kit was used. After 2 h of incubation, the absorbance was measured at 450 nm with a reference at 620 nm. Cell viability was calculated as a percentage of the control.

### 3.9. Anti-Inflammatory Activity

The mouse macrophage RAW264.7 cell line was purchased from the American Type Culture Collection. Cells were cultured in DMEM supplemented with 10% fetal bovine serum (FBS), 100 units/mL penicillin, and 100 μg/mL streptomycin, and incubated at 37 °C in a humidified atmosphere with 5% CO_2_. Cell viability was measured using the Ez-Cytox cell viability detection kit. Cells were incubated in 96-well plates at a concentration of 2500 cells per well for 24 h and treated with the indicated concentrations of compounds **1**–**3** for 24 h. Next, Ez-Cytox reagents were added to each well. The optical density at 450 nm was measured after 1 h using a microplate reader (PowerWave XS; Bio-Tek Instruments, Winooski, VT, USA) to estimate cell viability. In a separate experiment, RAW264.7 cells were incubated in 96-well plates at a concentration of 30,000 cells per well for 24 h. After serum starvation for 12 h, cells were pretreated with compounds **1**–**3** for 1 h and then stimulated with 1 µg/mL of LPS for 24 h. The absorbance of culture media in a Griess reaction was measured at 540 nm using a PowerWave XS microplate reader to estimate the NO level.

### 3.10. Measurement of Melanin Content

The mouse melanoma cell line, B16F10, was purchased from the American Type Culture Collection. Cells were cultured in DMEM supplemented with 10% FBS, 100 units/mL penicillin, and 100 μg/mL streptomycin, and incubated at 37 °C in a humidified atmosphere with 5% CO_2_. B16F10 cells were incubated in 6 cm culture dishes at 250,000 cells per well in DMEM supplemented with 10% FBS, 100 μg/mL streptomycin, and 100 U/mL penicillin for 24 h. The cells were washed twice with phosphate-buffered saline (PBS) and then stimulated by IBMX and incubated with different concentrations of compounds **1**–**3** or kojic acid (positive control) in phenol red-free RPMI medium supplemented with 10% FBS, 100 units/mL penicillin, and 100 μg/mL streptomycin for 24 h and 48 h. The absorbance of the culture medium was measured at 405 nm using a PowerWave XS microplate reader to estimate melanin content. Results are expressed as fold-change compared to the control (cells not stimulated by IBMX).

### 3.11. Statistical Analysis

All data including cell viability, NO, and melanin productions were presented as the average value and standard deviation (SD). All the assays were done in triplicateand were repeated at least three times. In this study, only a few numbers of repetitions of each cell experiment were included, thus the non-parametric analysis method was adopted for statistical analysis. The Kruskall–Wallis test was used for the statistical analysis of each variable. The SPSS statistical package was used for all analyses (International Business Machines Corporation (IBM) SPSS statistics version 21, Boston, MA, USA). Statistical significance was considered at a *p*-value lower than 0.05.

## 4. Conclusions

Our report provides a chemical analysis of microbial isolates from a fungus-growing termite. Three new cyclic tripeptides, natalenamides A–C (**1**–**3**), were isolated and structurally characterized from the culture broth of the fungus-growing -termite-associated *Actinomadura* sp. RB99 using an LC-UV/MS-based dereplication method. All the isolated compounds were evaluated for biological activities using cell-based assays. Compounds **1** and **2** exhibited weak cytotoxicity against HepG2 and HeLa/A549 cells, respectively. None of the isolated compounds showed significant inhibitory effects on NO production in LPS-stimulated RAW264.7 cells. Compound **3** exhibited significant inhibitory effects on IBMX-mediated melanin synthesis in a dose-dependent manner. In fact, the effect was similar to than that of kojic acid, which is used as a cosmetic material with skin-whitening effects.

## Figures and Tables

**Table 1:** ^1^H (800 MHz) and ^13^C (200 MHz) NMR data of compounds **1**–**3** in *d*_4_-MeOH. ^a,b^.

1			2			3		
Position	*δ*_C_	*δ*_H_ (*J* in Hz)	Position	*δ*_C_	*δ*_H_ (*J* in Hz)	Position	*δ*_C_	*δ*_H_ (*J* in Hz)
1	60.4 d	4.16 d (7.5)	1	53.1 d	4.42 m	1	56.3 d	4.53 dd (7.5, 5.0)
2	32.1 d	2.02 m	2	42.4 t	1.63 m	2a	38.5 t	3.02 dd (14.0, 7.5)
3	18.9 q	0.91 d (7.0)	3	26.2 d	1.69 m	2b	-	3.24 dd (14.0, 5.0)
4	19.9 q	0.92 d (7.0)	4	22.1 q	0.92 d (6.5)	3	138.9 s	-
5	174.8 s	-	5	23.7 q	0.95 d (6.5)	4/8	130.7 d	7.21 m
6	58.0 d	4.20 dd (8.5, 4.5)	6	174.9 s	-	5/7	129.7 d	7.23 m
7a	27.0 t	1.95 m	7	58.1 d	4.09 dd (9.0, 4.5)	6	127.8 d	7.17 m
7b		2.48 m	8a	26.9 t	1.82 m	9	175.0 s	-
8a	30.6 t	2.27 m	8b	-	2.34 m	10	57.9 d	4.04 dd (9.0, 4.5) 8
8b		2.36 m	9a	30.3 t	2.16 m	11a	26.6 t	1.74 m
9	181.8 s	-	9b	-	2.19 m	11b	-	2.29 m
10	173.3 s	-	10	181.6 s	-	12	30.0 t	2.15 m
11	55.6 d	4.63 dd (8.0, 5.0)	11	173.4 s	-	13	181.7 s	-
12a	38.8 t	2.97 dd (14.0, 8.0)	12	55.8 d	4.72 dd (10.0, 5.0)	14	174.0 s	-
12b	-	3.20 dd (14.0, 5.0)	13a	38.9 t	2.89 dd (14.0, 10.0)	15	55.8 d	4.63 dd (10.0 4.5)
13	138.8 s	-	13b	-	3.23 dd (14.0, 5.0)	16a	38.4 t	2.80 dd (14.0, 10.0)
14/18	130.6 d	7.23 m	14	138.6 s	-	16b	-	3.23 dd (14.0, 4.5)
15/17	129.5 d	7.24 m	15/19	130.5 d	7.21 m	17	138.4 s	-
16	127.8 d	7.18 m	16/18	129.4 d	7.25 m	18/22	130.2 d	7.21 m
19	173.2 s	-	17	127.6 d	7.19 m	19/21	129.3 d	7.23 m
-	-	-	20	173.3 s	-	20	127.4 d	7.17 m
-	-	-	-	-	-	23	173.8 s	-

^a^ Coupling constants (*J*, in parentheses) are in Hz, chemical shifts (*δ*) are in ppm; ^b 13^C NMR data were assigned based on the HSQC and HMBC experiment.

## Supplementary Materials

The following are available online: Figure S1. HR-ESIMS data of **1**; Figure S2. ^1^H NMR spectrum of **1** (CD_3_OD, 800 MHz); Figure S3. ^1^H-^1^H COSY spectrum of **1** (CD_3_OD); Figure S4. HSQC spectrum of **1** (CD_3_OD); Figure S5. HMBC spectrum of **1** (CD_3_OD); Figure S6. HR-ESIMS data of **2**; Figure S7. ^1^H NMR spectrum of **2** (CD_3_OD, 800 MHz); Figure S8. ^1^H-^1^H COSY spectrum of **2** (CD_3_OD); Figure S9. HSQC spectrum of **2** (CD_3_OD); Figure S10. HMBC spectrum of **2** (CD_3_OD); Figure S11. HR-ESIMS data of **3**; Figure S12. ^1^H NMR spectrum of **3** (CD_3_OD, 800 MHz); Figure S13. ^1^H-^1^H COSY spectrum of **3** (CD_3_OD); Figure S14. HSQC spectrum of **3** (CD_3_OD); Figure S15. HMBC spectrum of **3** (CD_3_OD); Figure S16. Retention times of the l-FDAA derivatized amino acids of standards; Figure S17. Retention times of the l-FDAA derivatized amino acids from compound **1**; Figure S18. Retention times of the l-FDAA derivatized l-Leu from compound **2**; Figure S19. Stimulation of production of compounds **1**–**3**; Figure S20. Expanded key HMBC correlations of **1**; Figure S21. Expanded key HMBC correlations of **2**; Figure S22. Expanded key HMBC correlations of **3**; Figure S23. LC/MS analysis of compounds **1**–**3**; Figure S24. Inhibitory effects of three fatty acid analogues on melanin content in B16F10 cells; Figure S25. Neighbor-joining tree based on almost complete 16S rRNA gene sequences.
