# Supplementary material for: Natalenamides A–C, Cyclic Tripeptides from the Termite-Associated Actinomadura sp. RB99
Source: Molecules. 2018 Nov 16;23(11):3003. doi: 10.3390/molecules23113003 (PMC6278286; doi:10.3390/molecules23113003)

Supporting Information

Natalenamides A-C, Cyclic Tripeptides from the Termite-associated *Actinomadura* sp. RB99

Seoung Rak Lee [1], Dahae Lee [1], Jae Sik Yu [1], René Benndorf [2], Sullim Lee [3], Dong-Soo Lee [4], Jungmoo Huh [5], Z. Wilhelm de Beer [6], Yong Ho Kim [7], Christine Beemelmanns [2], Ki Sung Kang [4], and Ki Hyun Kim [1,*]

[1] School of Pharmacy, Sungkyunkwan University, Suwon 16419, Republic of Korea; davidseoungrak@gmail.com (S.R.L.); pjsldh@naver.com (D.L.); jsyu@bu.edu (J.S.Y.)
[2] Leibniz Institute for Natural Product Research and Infection Biology – Hans-Knöll-Institute, Beutenbergstraße 11a, 07745 Jena, Germany; rene.benndorf@hki-jena.de (R.B.); Christine.beemelmanns@hki-jena.de (C.B.)
[3] College of Bio-Nano Technology, Gachon University, Seongnam 13120, Republic of Korea; sullimlee@gachon.ac.kr (S.L.)
[4] College of Korean Medicine, Gachon University, Seongnam 13120, Republic of Korea; vet4animal@hotmail.com (D.-S.L.); kkang@gachon.ac.kr (K.S.K.)
[5] College of Pharmacy and Research Institute of Pharmaceutical Sciences, Seoul National University, Gwanak-gu, Seoul 08826, Republic of Korea; goodhjm112@snu.ac.kr (J.H.)
[6] Forestry and Agriculture Biotechnology Institute, University of Pretoria, Pretoria, South Africa; wilhelm.debeer@fabi.up.ac.za (Z.W.B.)
[7] SKKU Advanced Institute of Nanotechnology (SAINT), Sungkyunkwan University, Suwon 16419, Republic of Korea; yhkim94@skku.edu (Y.H.K.)

* Correspondence: khkim83@skku.edu; Tel.: +82-31-290-7700

Supporting Information Contents:

Figure S1. HR-ESIMS data of **1**……S3
Figure S2. ^{1}H NMR spectrum of **1** (CD_3OD, 800 MHz)……S4
Figure S3. ^{1}H-^{1}H COSY spectrum of **1** (CD_3OD)……S5
Figure S4. HSQC spectrum of **1** (CD_3OD)……S6
Figure S5. HMBC spectrum of **1** (CD_3OD)……S7
Figure S6. HR-ESIMS data of **2**……S8
Figure S7. ^{1}H NMR spectrum of **2** (CD_3OD, 800 MHz)……S9
Figure S8. ^{1}H-^{1}H COSY spectrum of **2** (CD_3OD)……S10
Figure S9. HSQC spectrum of **2** (CD_3OD)……S11
Figure S10. HMBC spectrum of **2** (CD_3OD)……S12
Figure S11. HR-ESIMS data of **3**……S13
Figure S12. ^{1}H NMR spectrum of **3** (CD_3OD, 800 MHz)……S14
Figure S13. ^{1}H-^{1}H COSY spectrum of **3** (CD_3OD)……S15
Figure S14. HSQC spectrum of **3** (CD_3OD)……S16
Figure S15. HMBC spectrum of **3** (CD_3OD)……S17
Figure S16. Retention times of the L-FDAA derivatized amino acids of standards……S18–25
Figure S17. Retention times of the L-FDAA derivatized amino acids from compound **1**……S26–28
Figure S18. Retention times of the L-FDAA derivatized L-Leu from compound **2**……S29
Figure S19. Stimulation of production of compounds **1–3**……S30
Figure S20. Expanded key HMBC correlations of **1**……S31–33
Figure S21. Expanded key HMBC correlations of **2**……S34–36
Figure S22. Expanded key HMBC correlations of **3**……S37–39
Figure S23. LC/MS analysis of compounds **1-3** ……S40–S41
Figure S24. Inhibitory effects of three fatty acid analogues on melanin content in B16F10 cells……S42
Structural elucidation of the impurities in compound 3……S43
Figure S25. Neighbor-joining tree based on almost complete 16S rRNA gene sequences……S44

Figure S1. HR-ESIMS data of **1**

Mass	Calc. Mass	mDa	PPM	DBE	Formula	i-FIT	i-FIT Norm	Fit Conf %	C	H	N	O	Na
398.1691	398.1692	-0.1	-0.3	8.5	C19 H25 N3 O5 Na	33.3	2.765	6.30	19	25	3	5	1
	398.1689	0.2	0.5	12.5	C17 H20 N9 O3	34.0	3.491	3.05	17	20	9	3	
	398.1694	-0.3	-0.8	5.5	C2 H16 N21 O4	38.7	8.203	0.03	2	16	21	4	
	398.1697	-0.6	-1.5	1.5	C4 H21 N15 O6 Na	38.7	8.151	0.03	4	21	15	6	1
	398.1683	0.8	2.0	7.5	C H13 N25 Na	37.9	7.404	0.06	1	13	25		1
	398.1681	1.0	2.5	0.5	C H20 N17 O8	39.4	8.843	0.01	1	20	17	8	
	398.1705	-1.4	-3.5	13.5	C20 H21 N7 O Na	33.4	2.898	5.51	20	21	7	1	1
	398.1676	1.5	3.8	7.5	C16 H24 N5 O7	34.6	4.113	1.64	16	24	5	7	

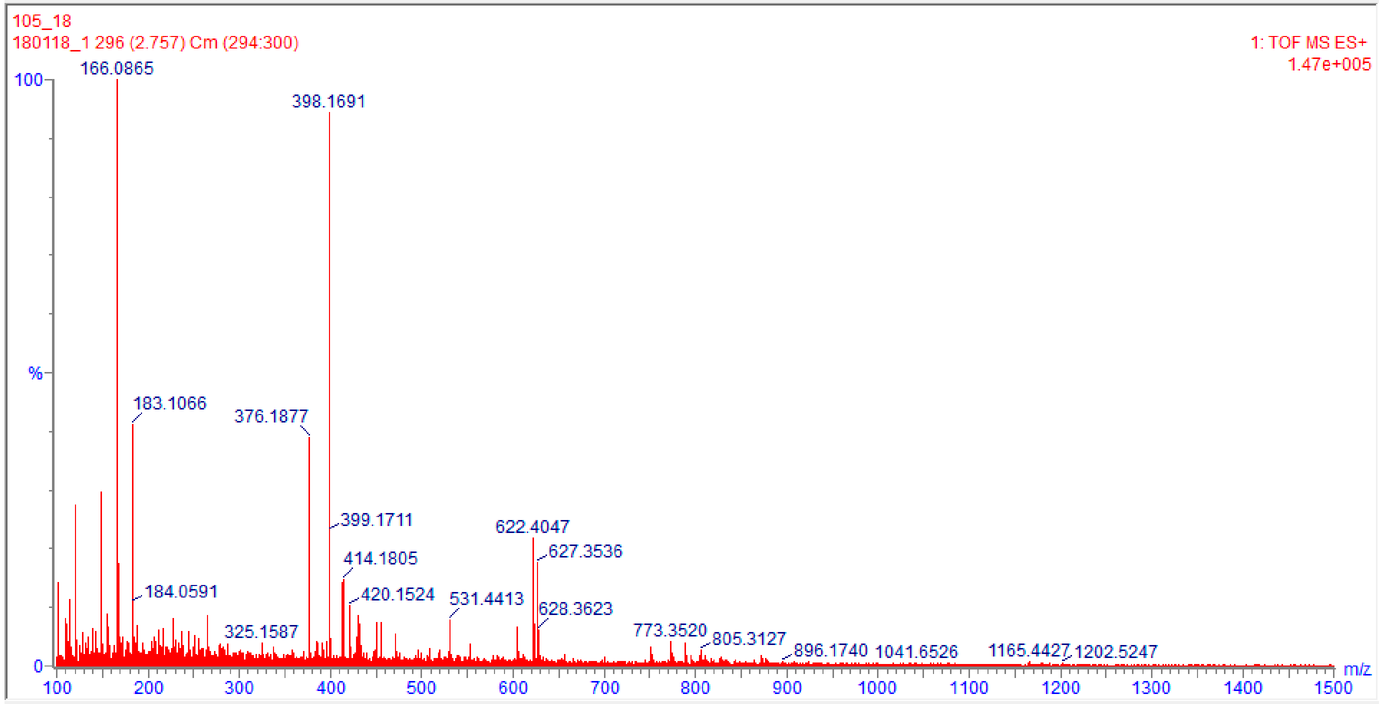

Figure S2. ^{1}H NMR spectrum of **1** (CD_3OD, 800 MHz)

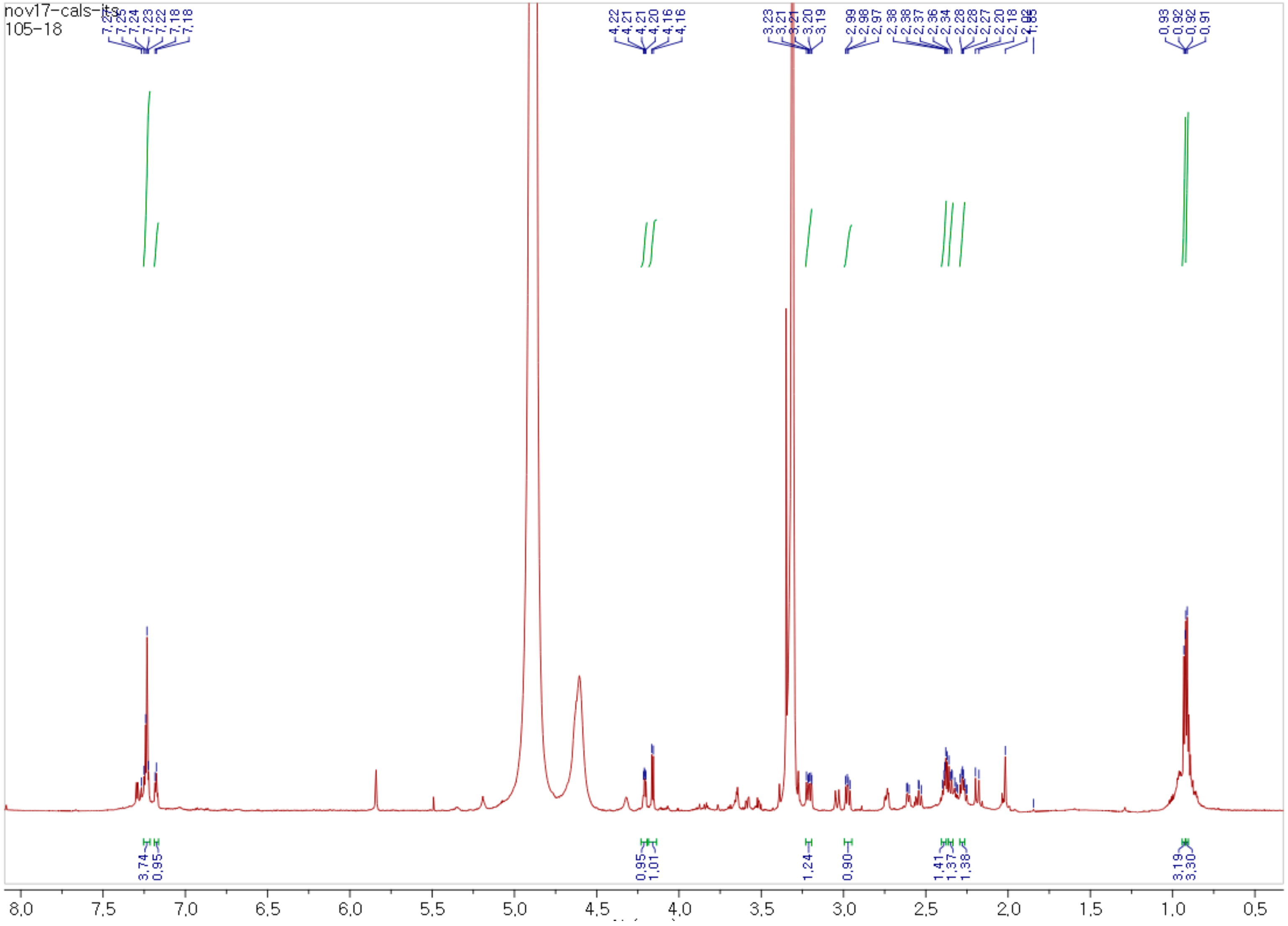

Figure S3. ^{1}H-^{1}H COSY spectrum of **1** (CD_3OD)

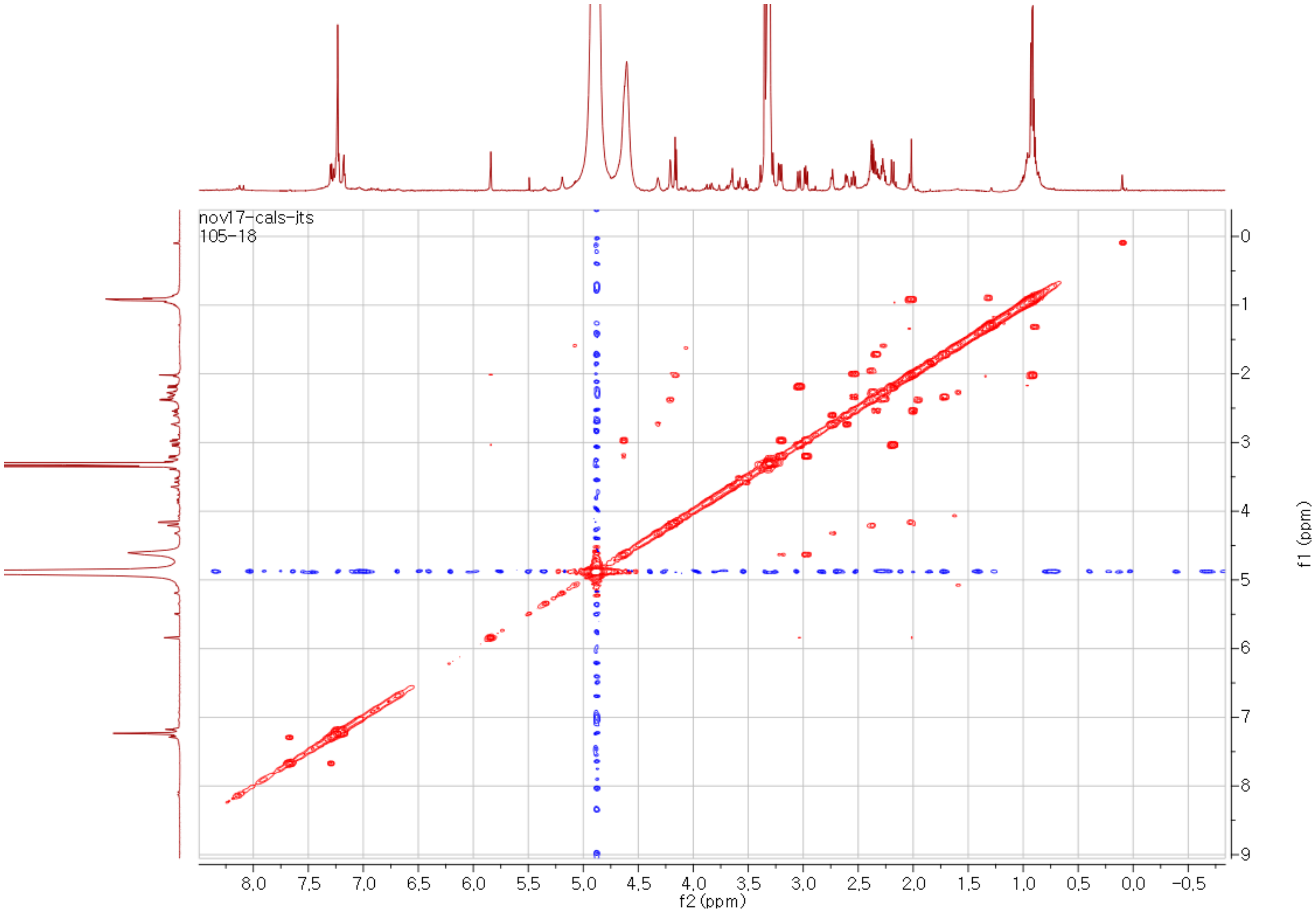

Figure S4. HSQC spectrum of **1** (CD_3OD)

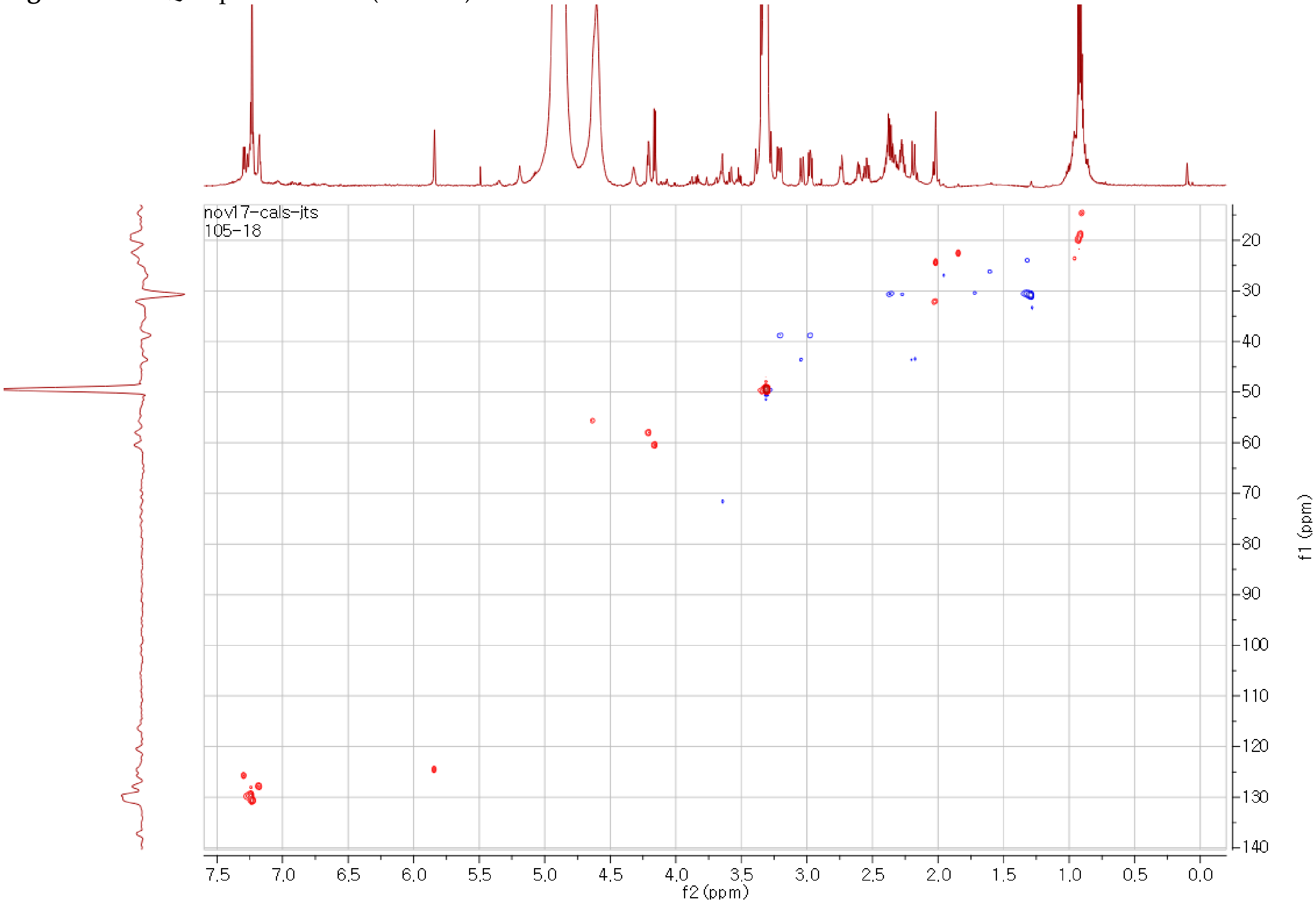

Figure S5. HMBC spectrum of **1** (CD_3OD)

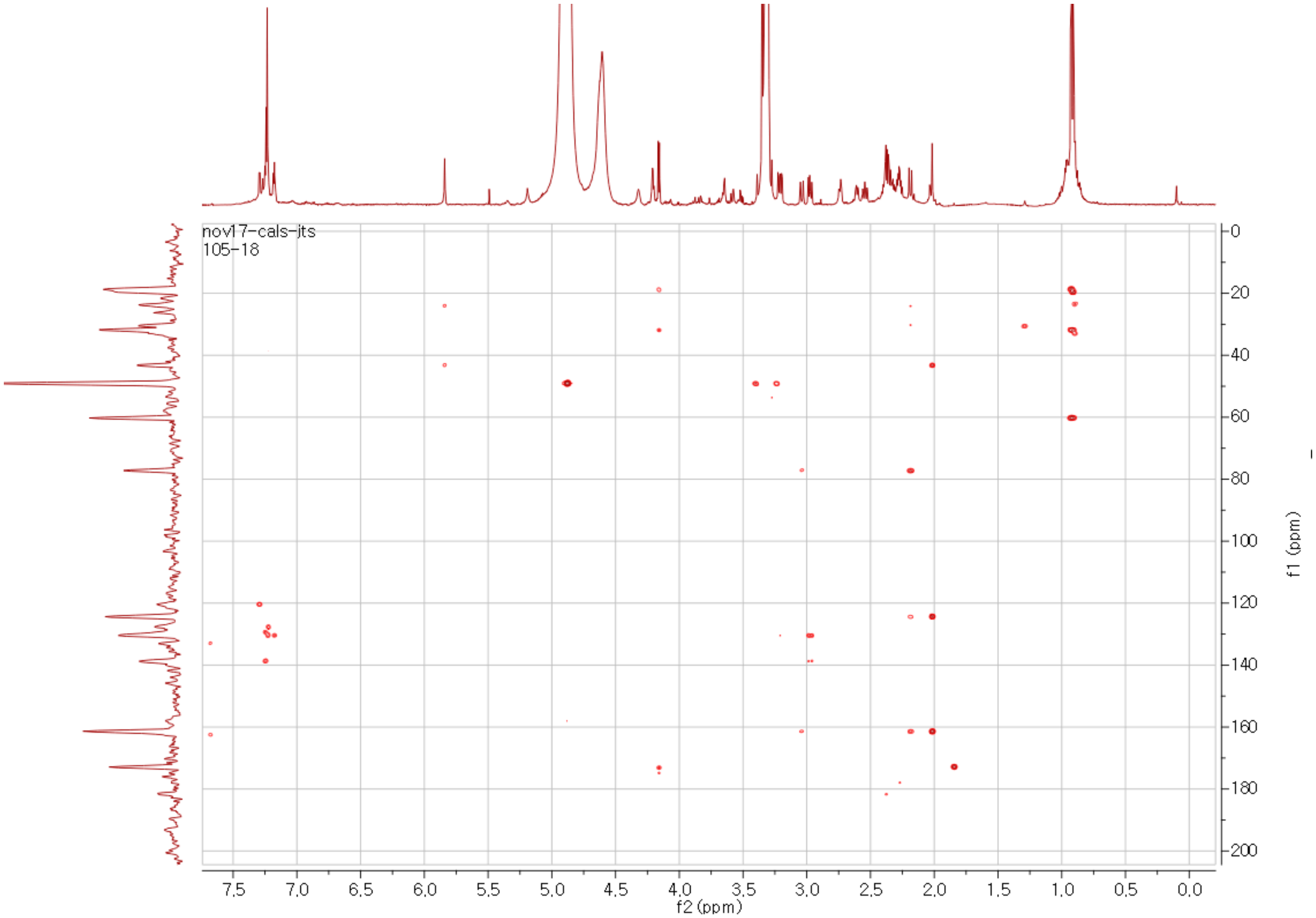

Figure S6. HR-ESIMS data of **2**

Mass	Calc. Mass	mDa	PPM	DBE	Formula	i-FIT	i-FIT Norm	Fit Conf %	C	H	N	O	Na
390.2032	390.2029	0.3	0.8	8.5	C20 H28 N3 O5	558.7	0.596	55.10	20	28	3	5	
	390.2005	2.7	6.9	5.5	C18 H29 N3 O5 Na	559.3	1.152	31.61	18	29	3	5	1
	390.2018	1.4	3.6	10.5	C19 H25 N7 O Na	561.2	3.110	4.46	19	25	7	1	1
	390.2045	-1.3	-3.3	9.5	C23 H29 N O3 Na	561.4	3.255	3.86	23	29	1	3	1
	390.2042	-1.0	-2.6	13.5	C21 H24 N7 O	562.0	3.881	2.06	21	24	7	1	
	390.2002	3.0	7.7	9.5	C16 H24 N9 O3	562.1	4.025	1.79	16	24	9	3	
	390.1989	4.3	11.0	4.5	C15 H28 N5 O7	563.0	4.911	0.74	15	28	5	7	
	390.2069	-3.7	-9.5	12.5	C25 H28 N O3	563.9	5.781	0.31	25	28	1	3	

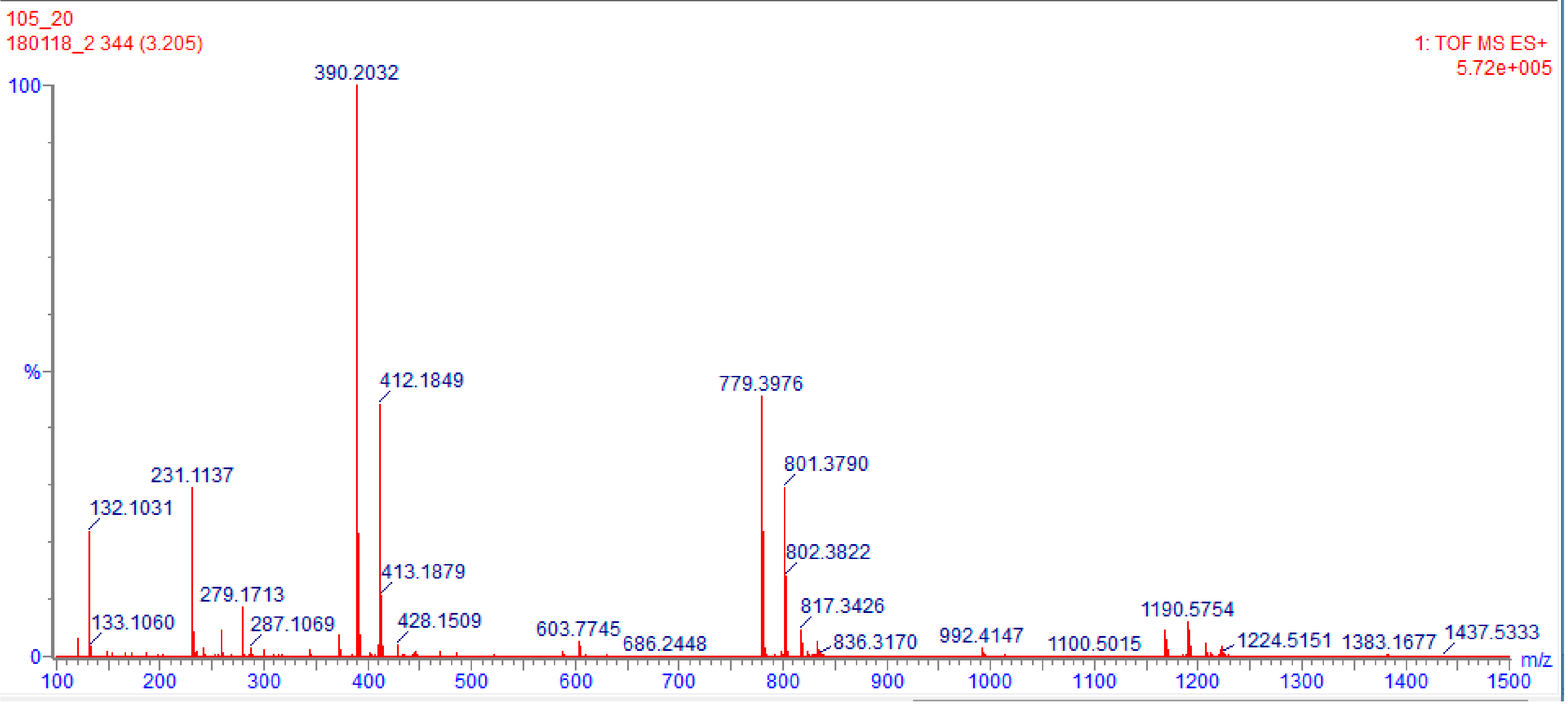

Figure S7. ^{1}H NMR spectrum of **2** (CD_3OD, 800 MHz)

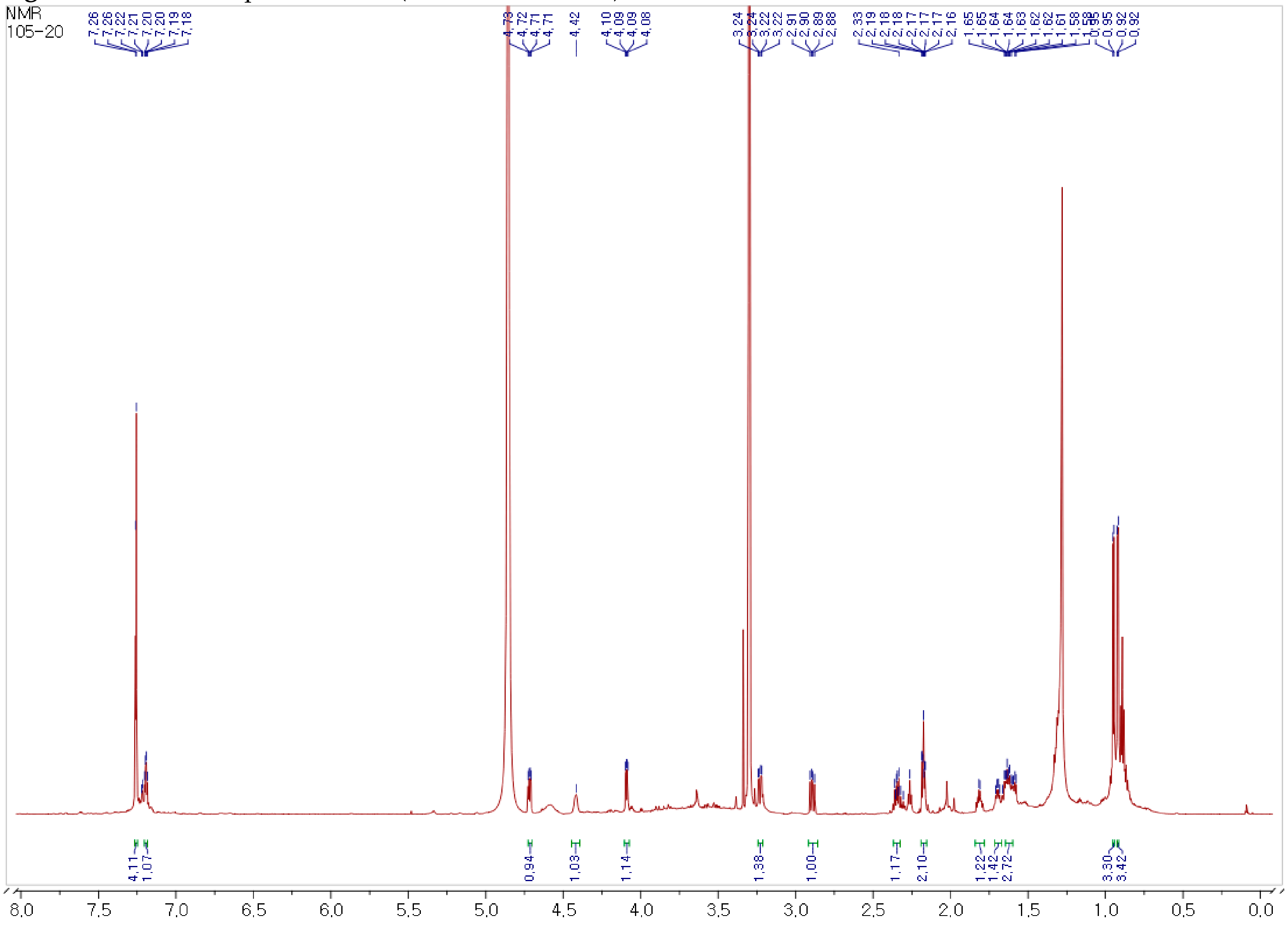

Figure S8. ^{1}H-^{1}H COSY spectrum of **2** (CD_3OD)

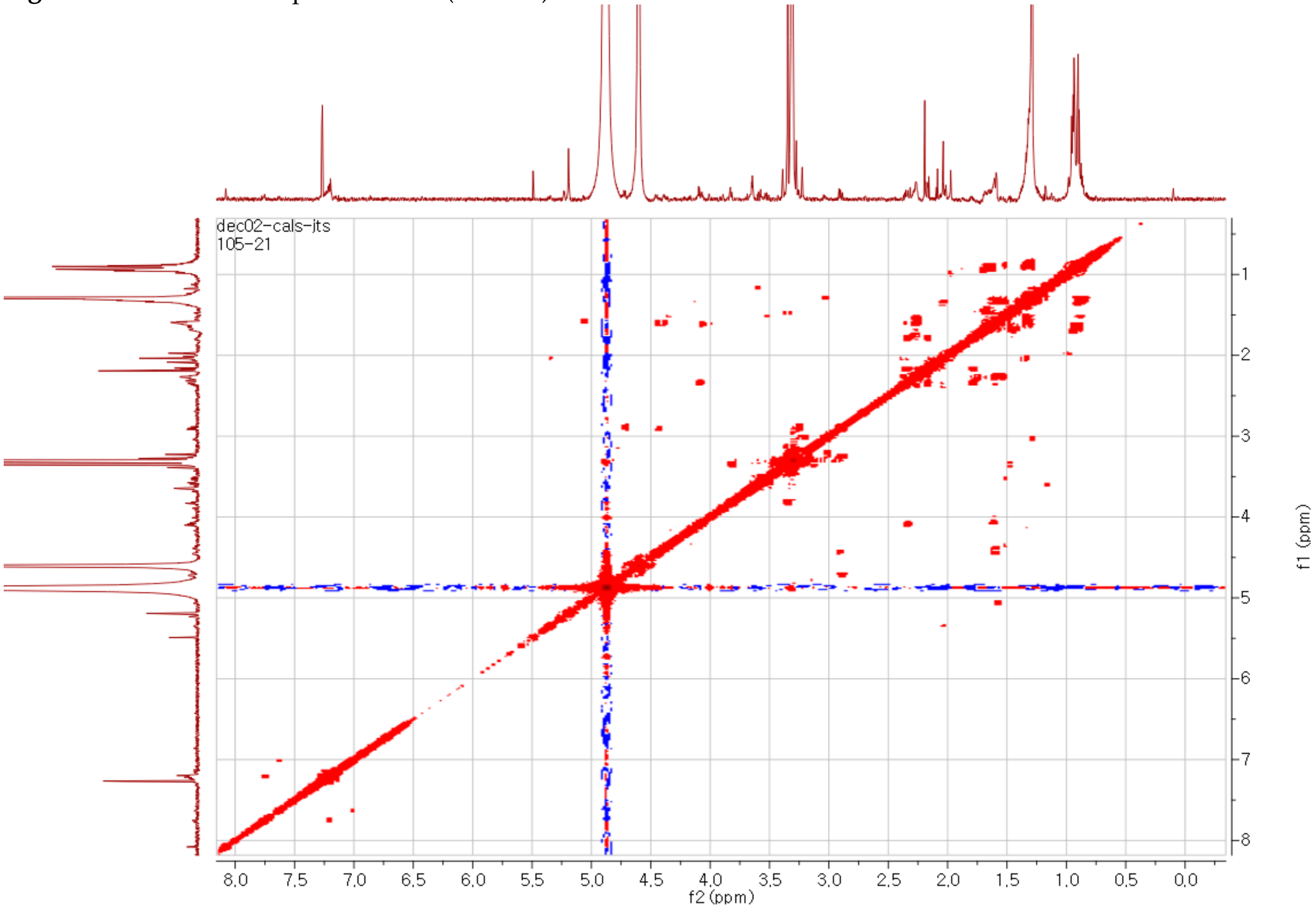

Figure S9. HSQC spectrum of **2** (CD_3OD)

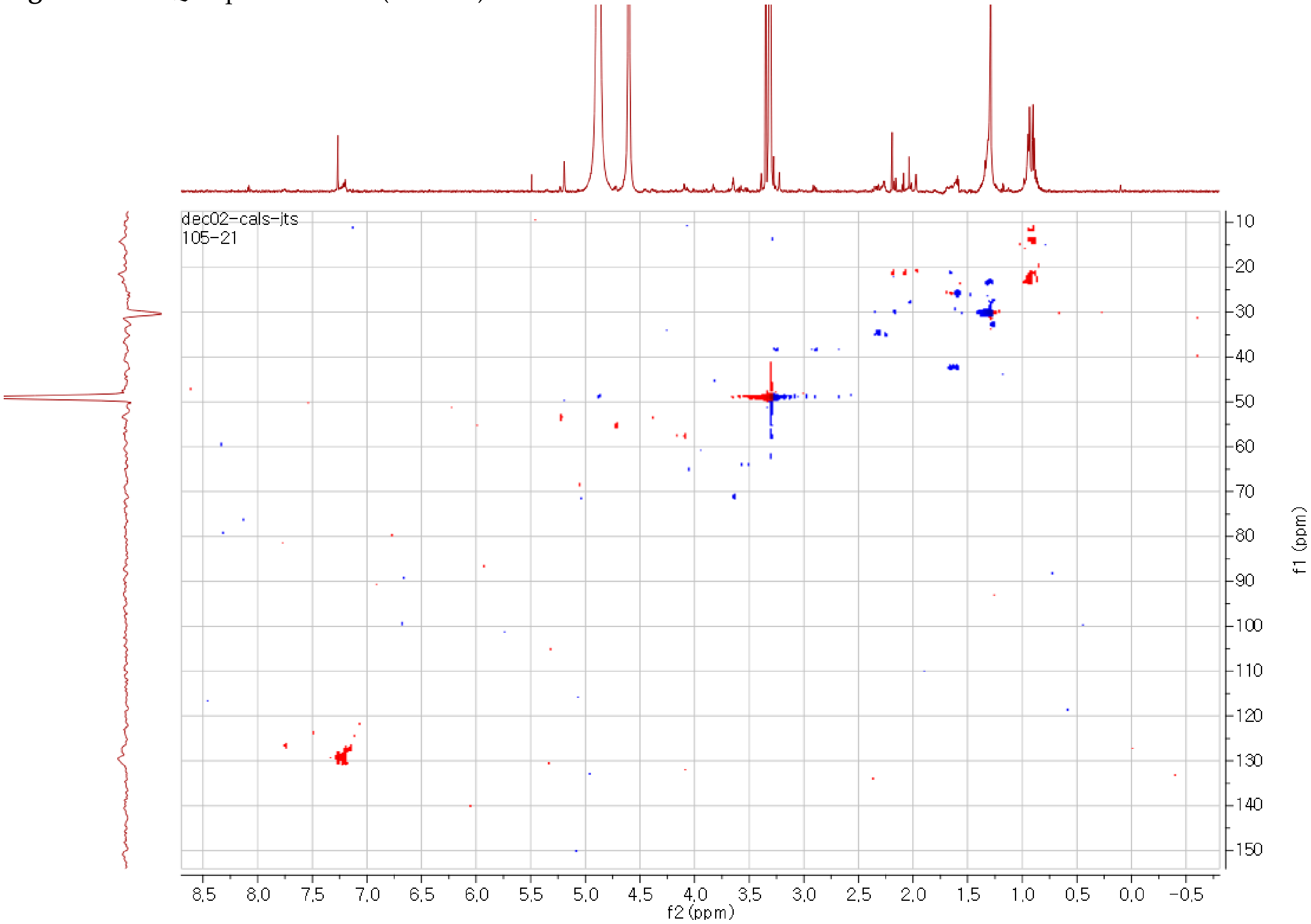

Figure S10. HMBC spectrum of **2** (CD_3OD)

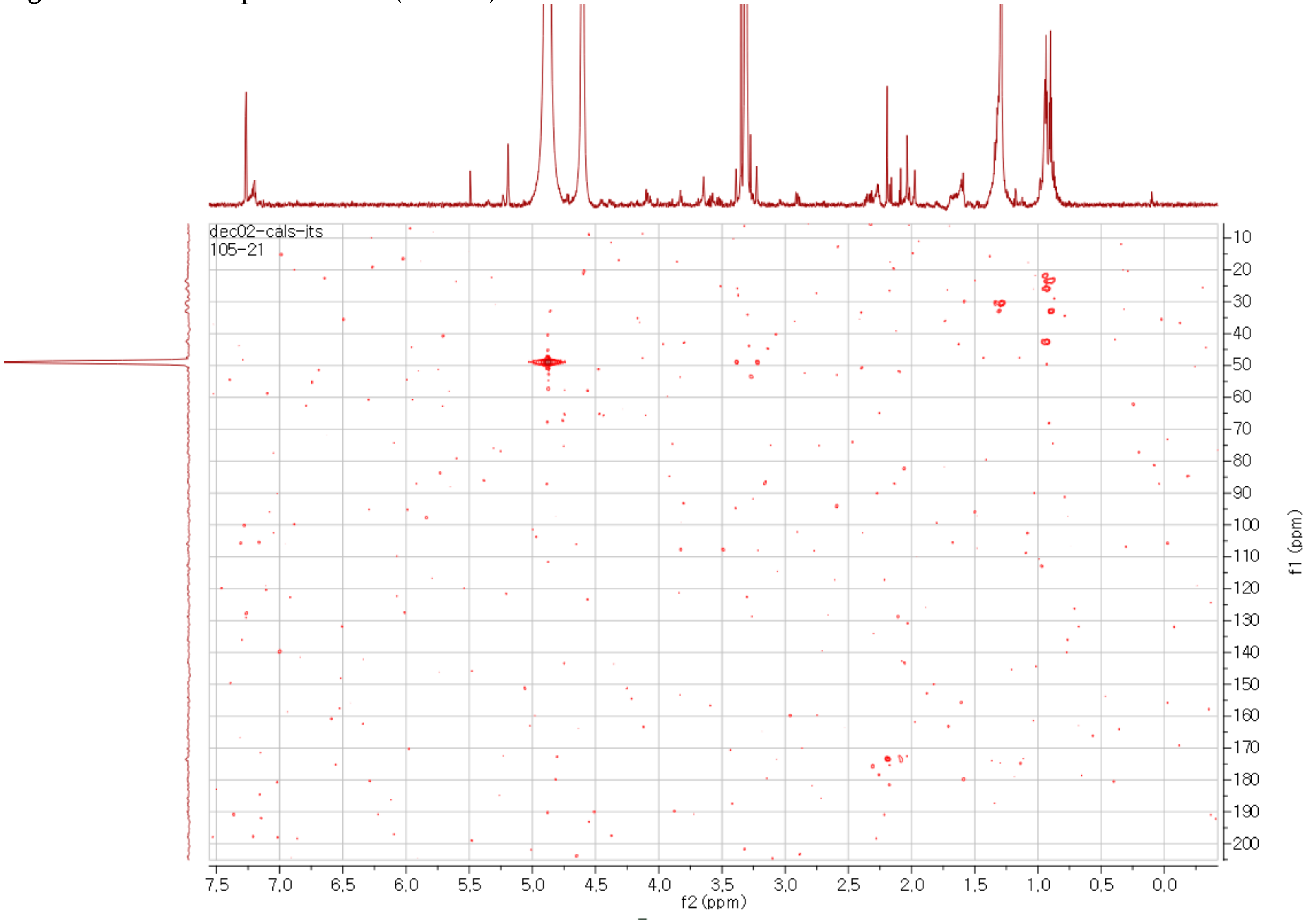

Figure S11. HR-ESIMS data of **3**

Mass	Calc. Mass	mDa	PPM	DBE	Formula	i-FIT	i-FIT Norm	Fit Conf %	C	H	N	O	Na
446.1694	446.1705	-1.1	-2.5	17.5	C24 H21 N7 O Na	223.7	0.369	69.12	24	21	7	1	1
	446.1689	0.5	1.1	16.5	C21 H20 N9 O3	225.2	1.846	15.79	21	20	9	3	
	446.1692	0.2	0.4	12.5	C23 H25 N3 O5 Na	225.4	2.086	12.42	23	25	3	5	1
	446.1716	-2.2	-4.9	15.5	C25 H24 N3 O5	227.6	4.269	1.40	25	24	3	5	
	446.1676	1.8	4.0	11.5	C20 H24 N5 O7	228.5	5.150	0.58	20	24	5	7	
	446.1729	-3.5	-7.8	20.5	C26 H20 N7 O	229.1	5.704	0.33	26	20	7	1	
	446.1665	2.9	6.5	13.5	C19 H21 N9 O3 Na	229.5	6.162	0.21	19	21	9	3	1
	446.1732	-3.8	-8.5	16.5	C28 H25 N O3 Na	230.7	7.359	0.06	28	25	1	3	1

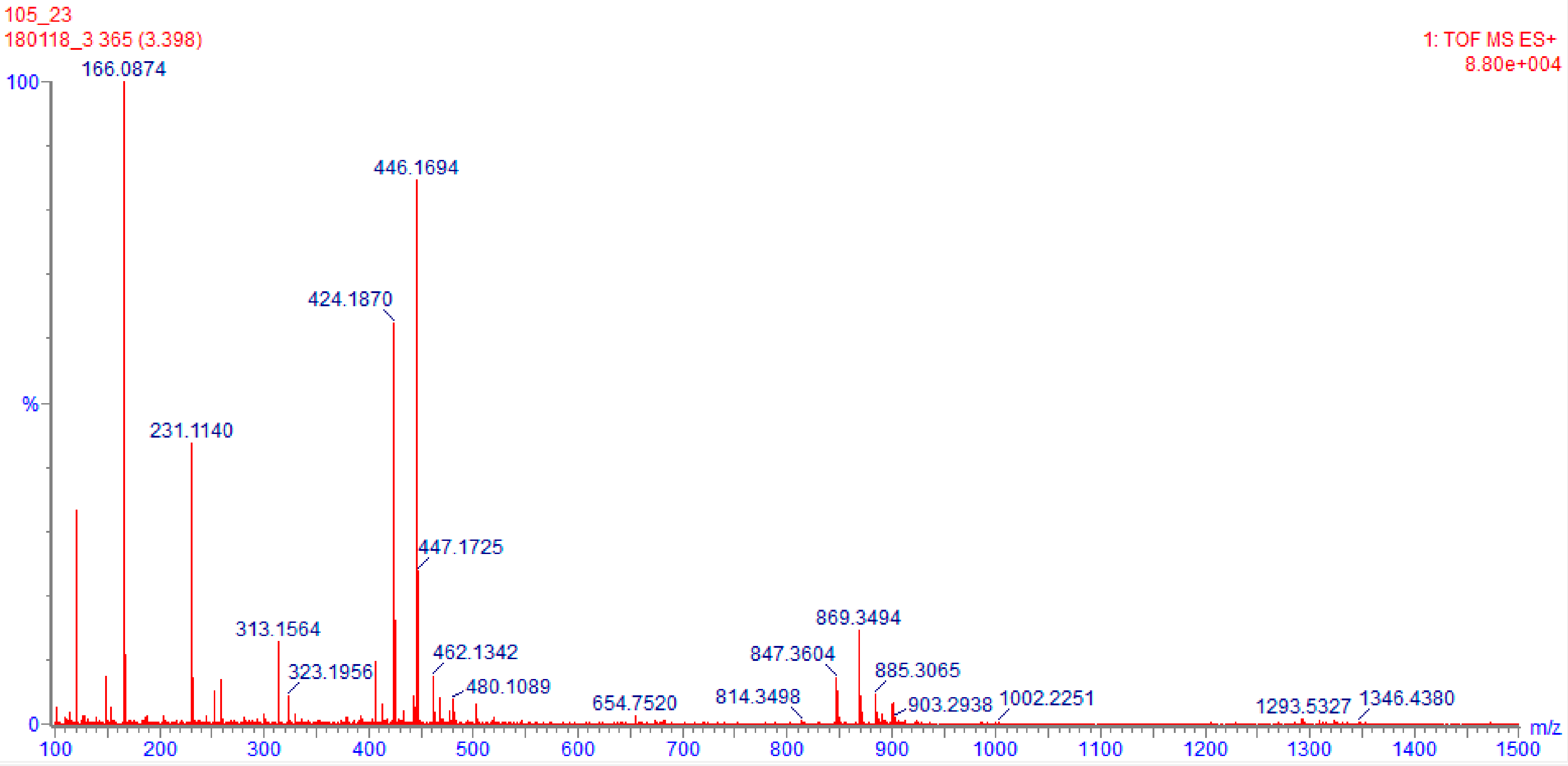

Figure S12. ^{1}H NMR spectrum of **3** (CD_3OD, 800 MHz)

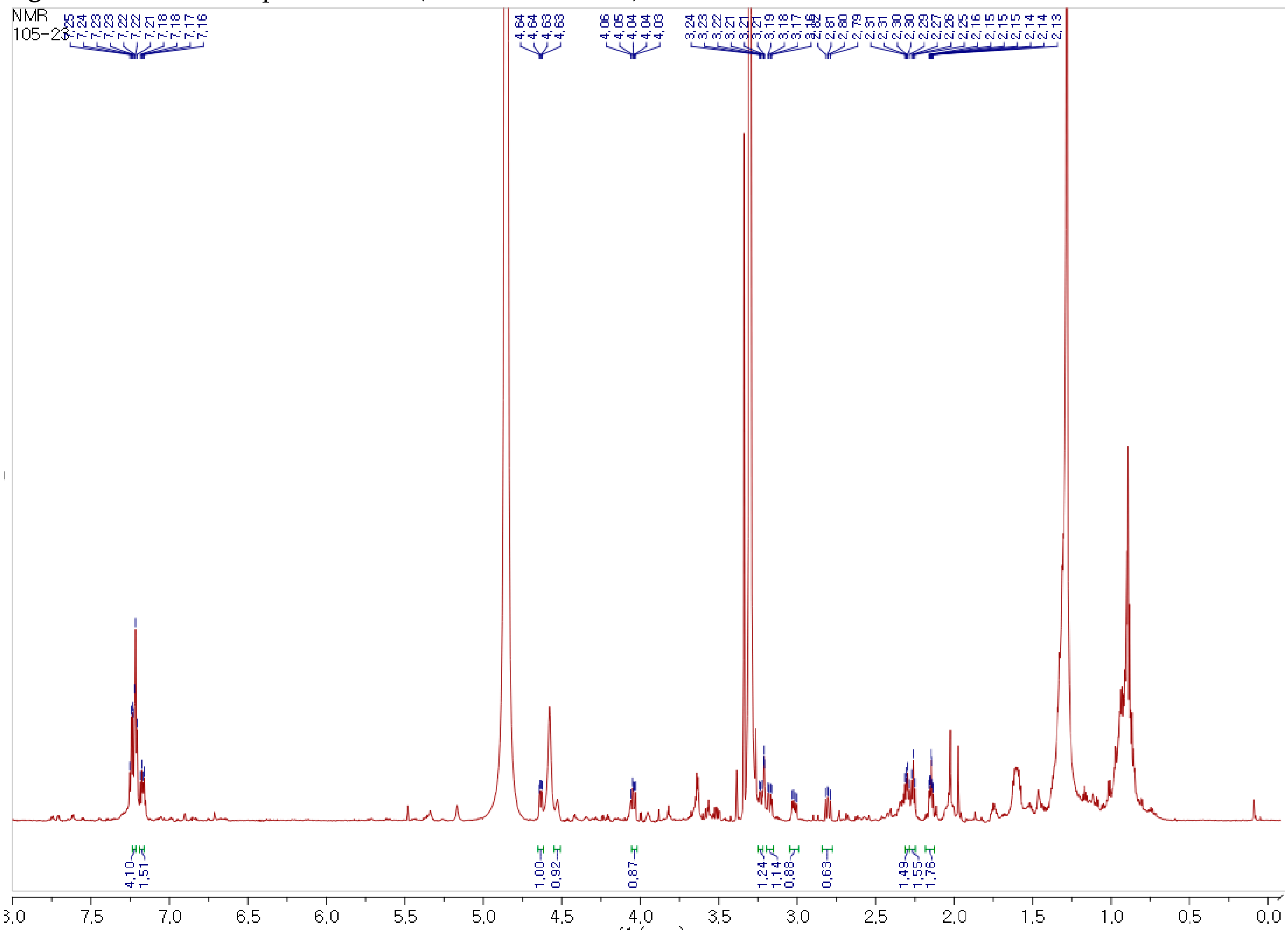

Figure S13. ^{1}H-^{1}H COSY spectrum of **3** (CD_3OD)

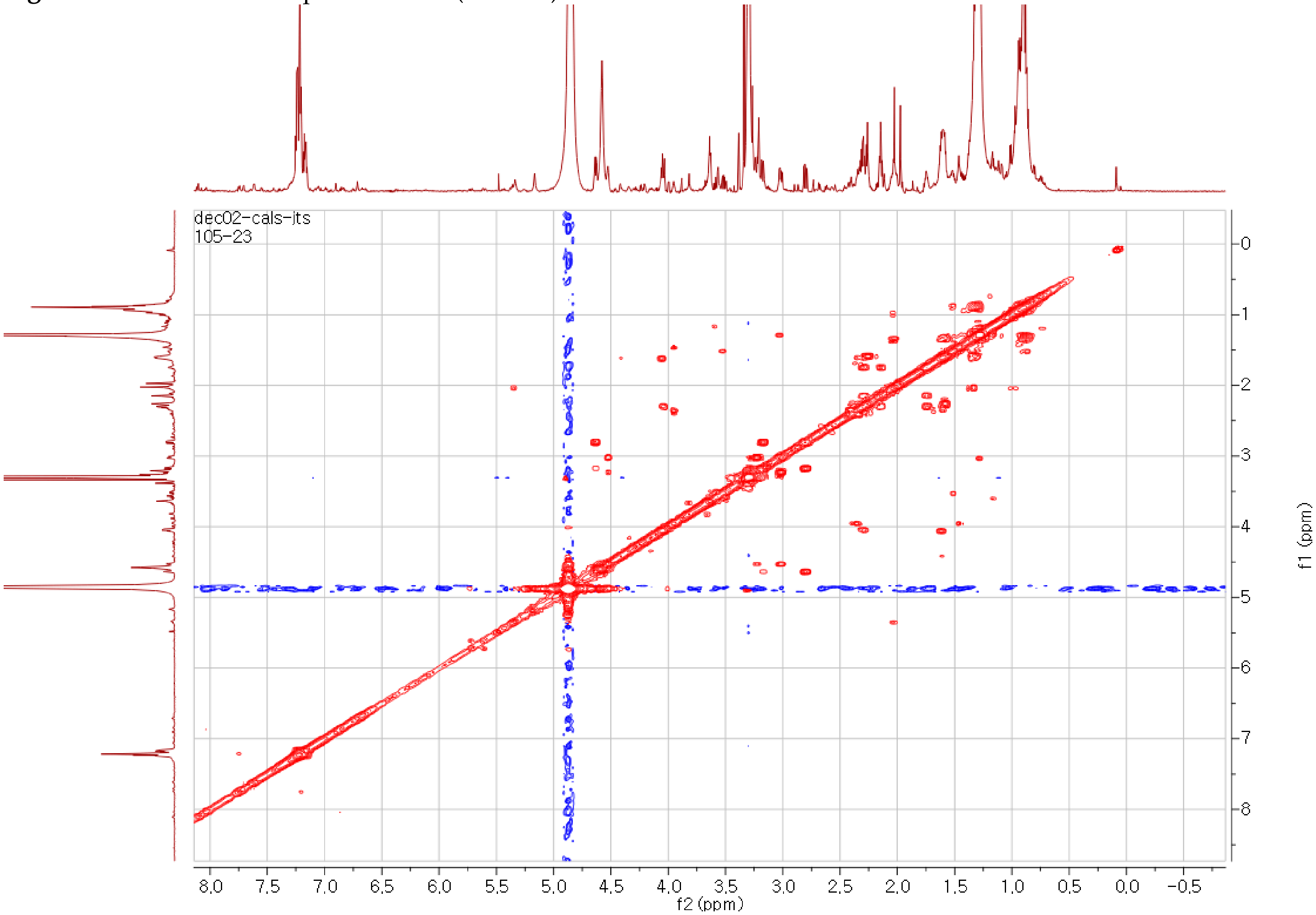

Figure S14. HSQC spectrum of **3** (CD_3OD)

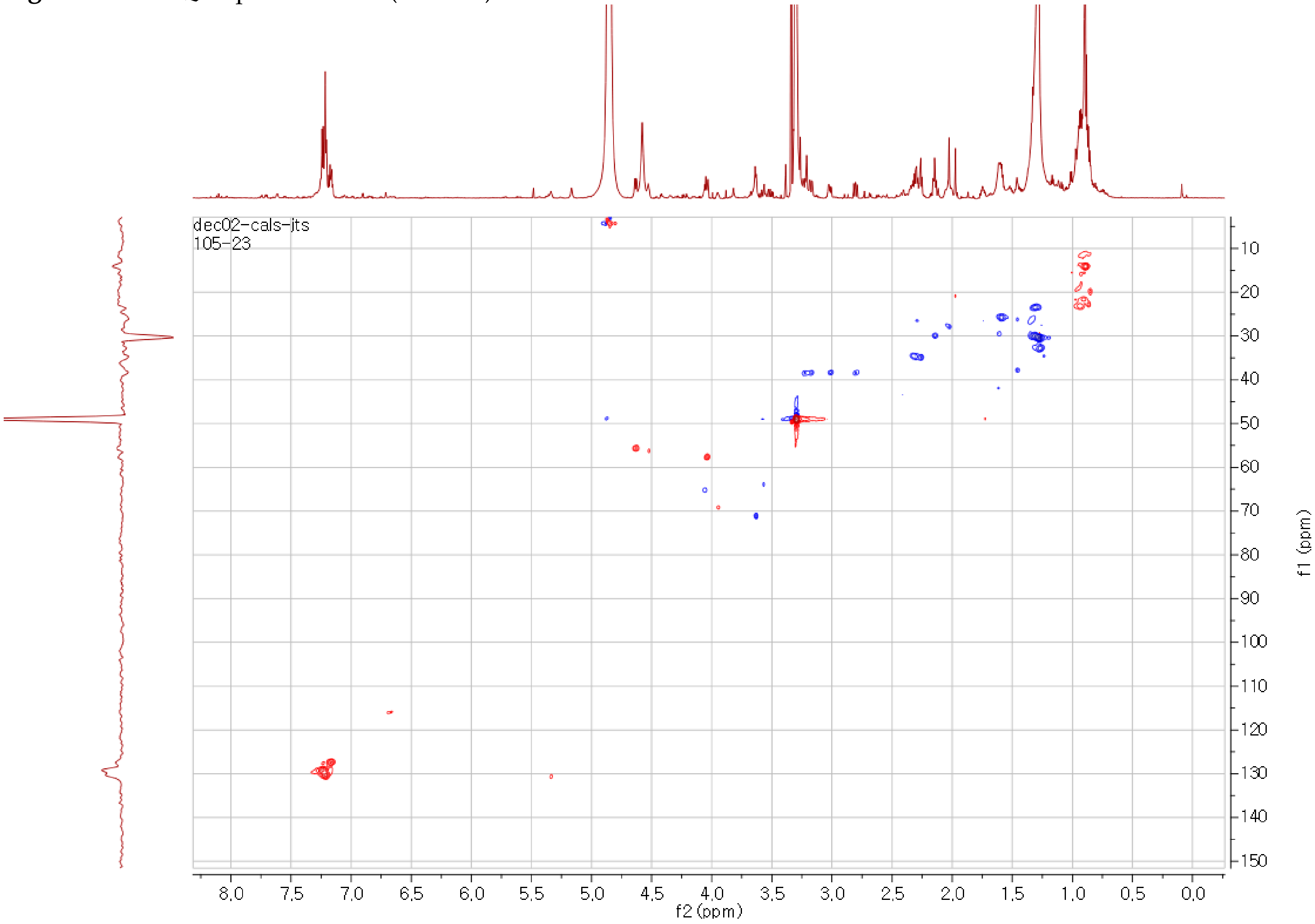

Figure S15. HMBC spectrum of **3** (CD_3OD)

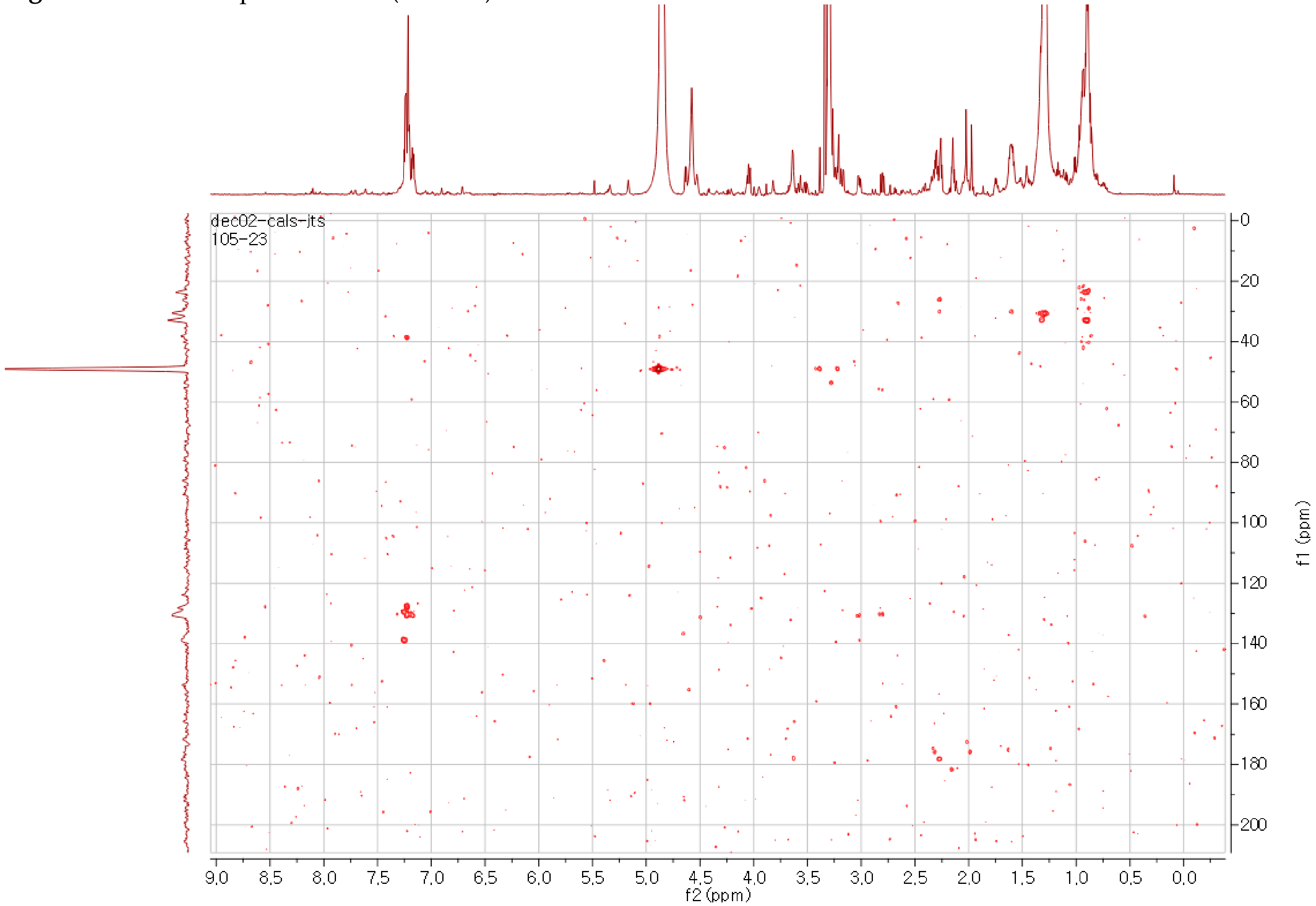

Figure S16. Retention times of the L-FDAA derivatized amino acids of standards

1) L-Glu

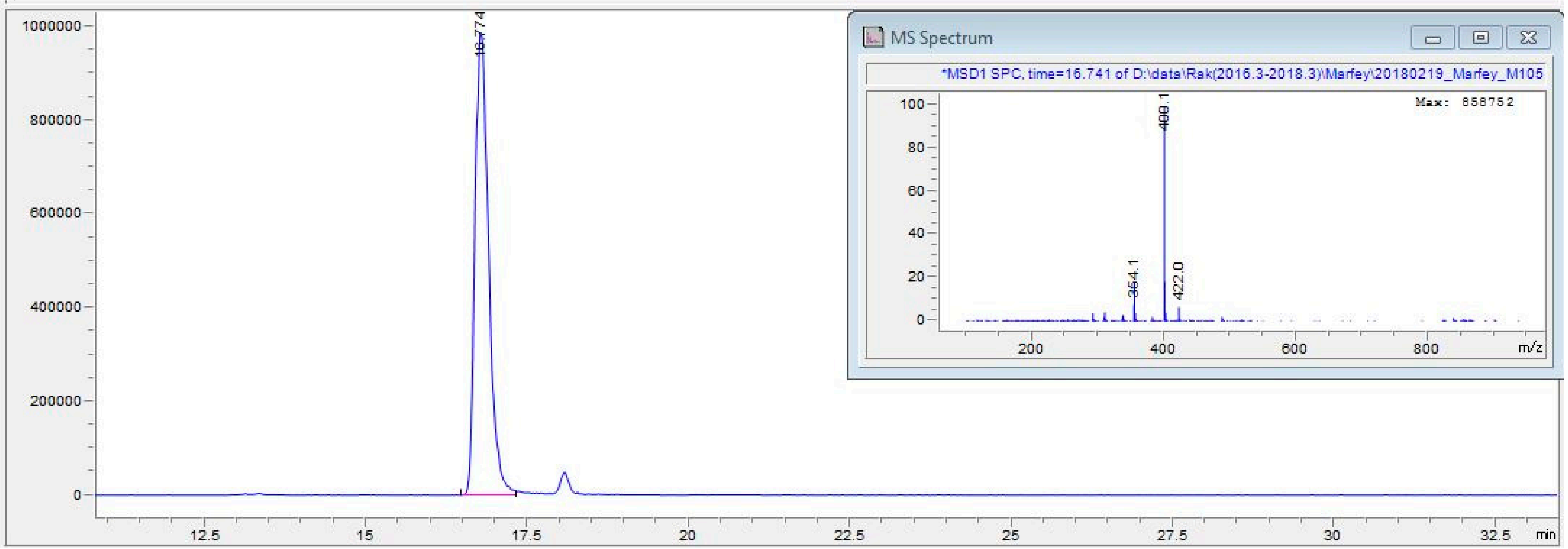

2) D-Glu

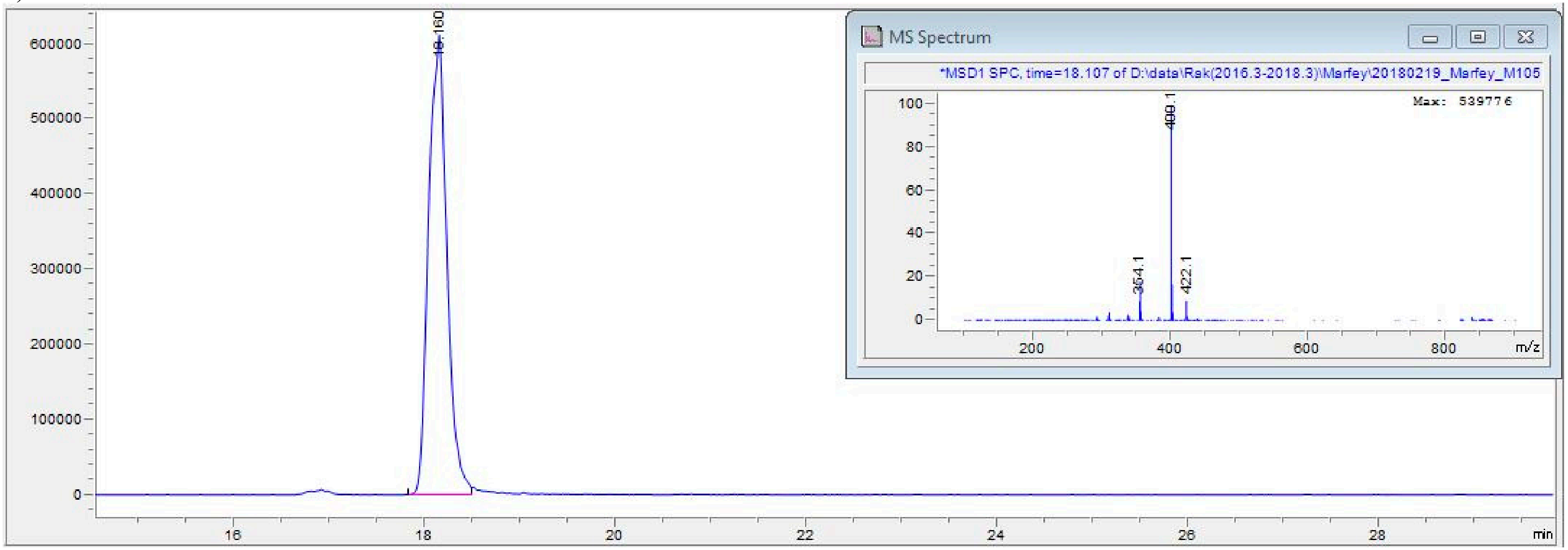

18.160
600000
500000
400000
300000
200000
100000
0
16
18
20
22
24
26
28
min
MS Spectrum
*MSD1 SPC, time=18.107 of D:\data\Rak(2016.3-2018.3)\Marfey\20180219_Marfey_M105
Max: 539776
400.1
354.1
422.1
100
80
60
40
20
0
200
400
600
800
m/z

3) L-Val

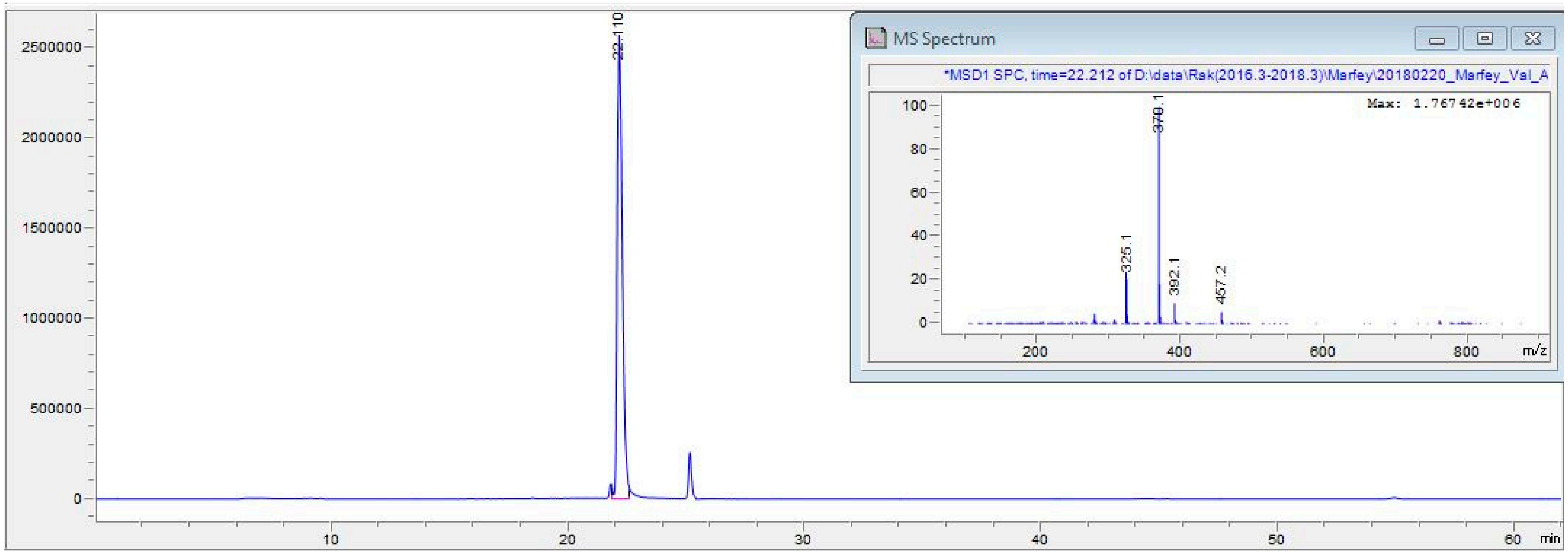

2500000
2000000
1500000
1000000
500000
0
22.110
10
20
30
40
50
60
min
MS Spectrum
*MSD1 SPC, time=22.212 of D:\data\Rak(2016.3-2018.3)\Marfey\20180220_Marfey_Val_A
Max: 1.76742e+006
100
80
60
40
20
0
370.1
325.1
392.1
457.2
200
400
600
800
m/z

4) D-Val

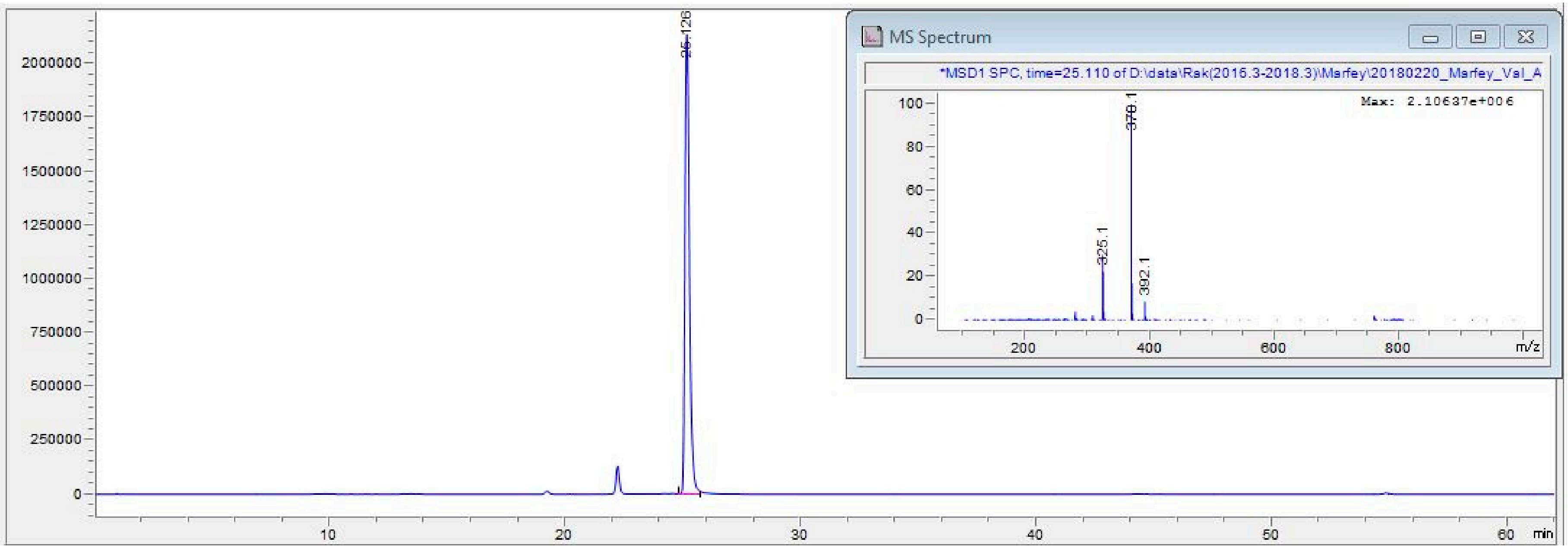

25.126
2000000
1750000
1500000
1250000
1000000
750000
500000
250000
0
10
20
30
40
50
60
min
MS Spectrum
*MSD1 SPC, time=25.110 of D:\data\Rak(2016.3-2018.3)\Marfey\20180220_Marfey_Val_A
Max: 2.10637e+006
100
80
60
40
20
0
370.1
325.1
392.1
200
400
600
800
m/z

5) L-Phe

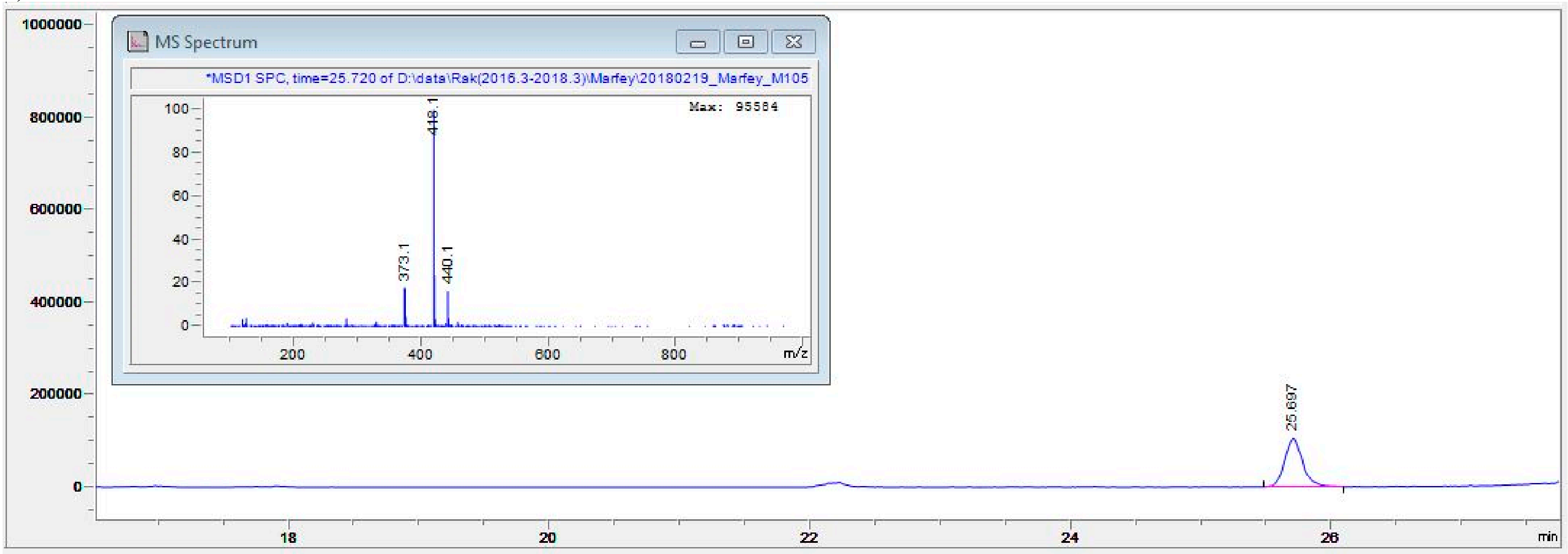
MS Spectrum
*MSD1 SPC, time=25.720 of D:\data\Rak(2016.3-2018.3)\Marfey\20180219_Marfey_M105
Max: 95584
418.1
373.1
440.1
m/z
25.697
min

6) D-Phe

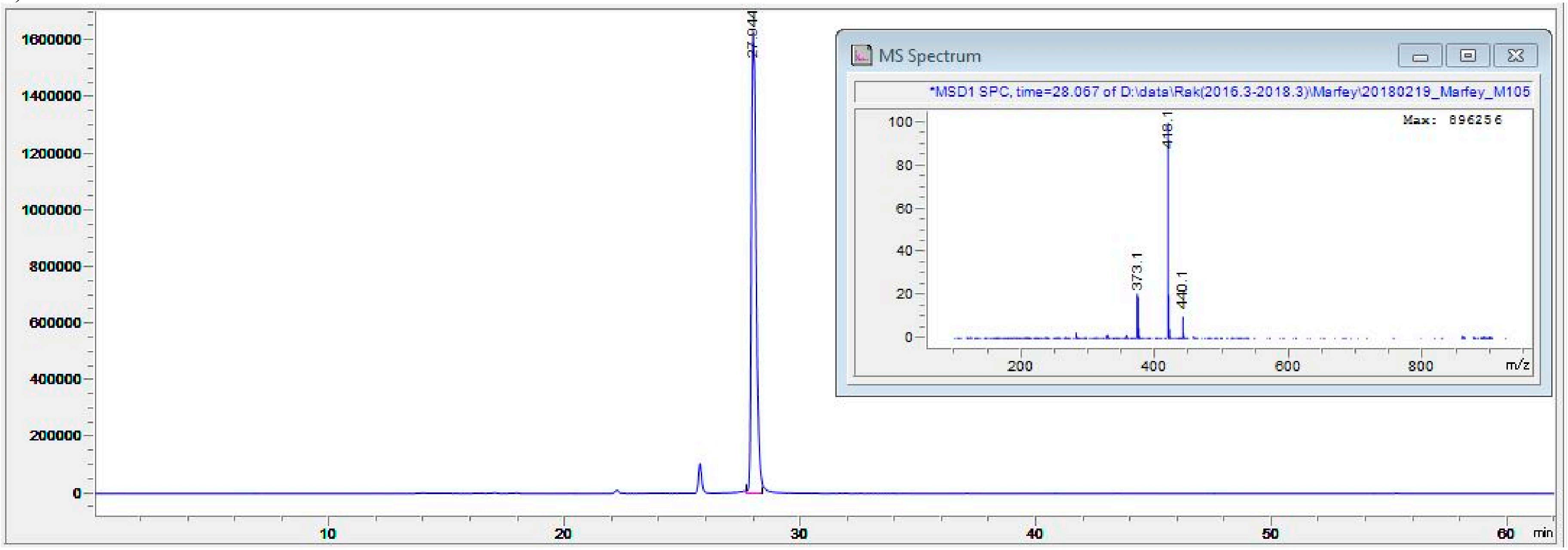

1600000
1400000
1200000
1000000
800000
600000
400000
200000
0
27.944
10
20
30
40
50
60
min
MS Spectrum
*MSD1 SPC, time=28.067 of D:\data\Rak(2016.3-2018.3)\Marfey\20180219_Marfey_M105
Max: 896256
100
80
60
40
20
0
418.1
373.1
440.1
200
400
600
800
m/z

7) L-Leu

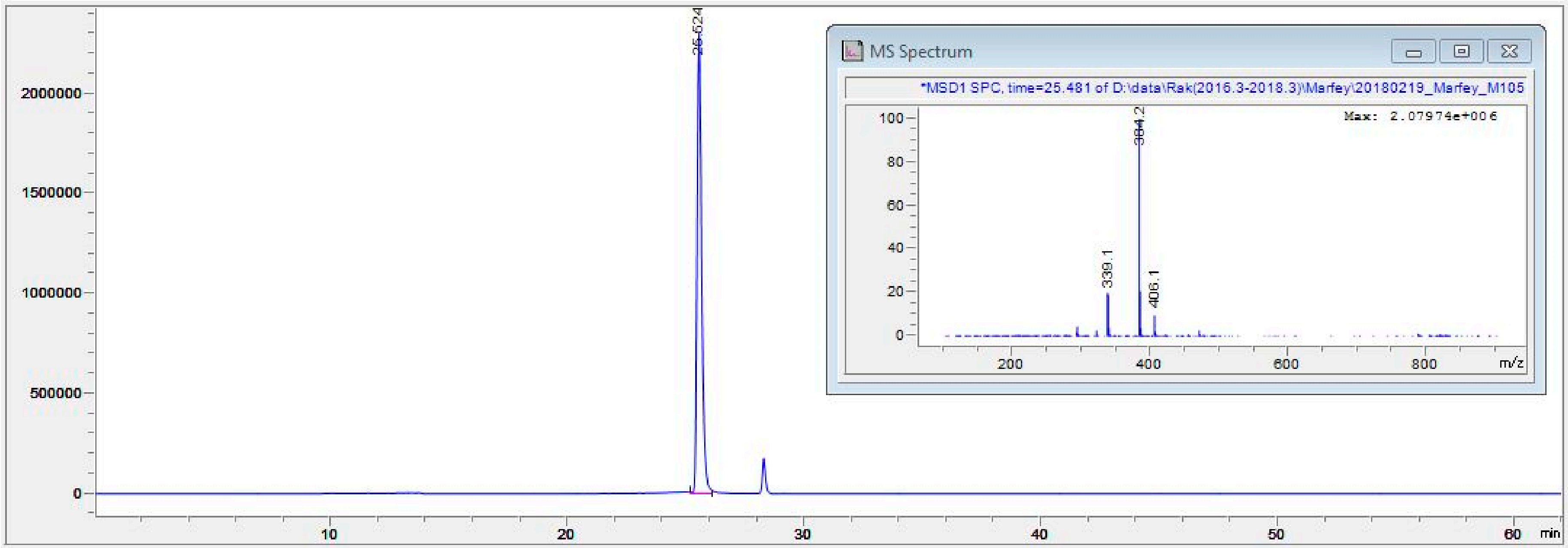

25.624
2000000
1500000
1000000
500000
0
10
20
30
40
50
60
min
MS Spectrum
*MSD1 SPC, time=25.481 of D:\data\Rak(2016.3-2018.3)\Marfey\20180219_Marfey_M105
Max: 2.07974e+006
100
80
60
40
20
0
339.1
384.2
406.1
200
400
600
800
m/z

8) D-Leu

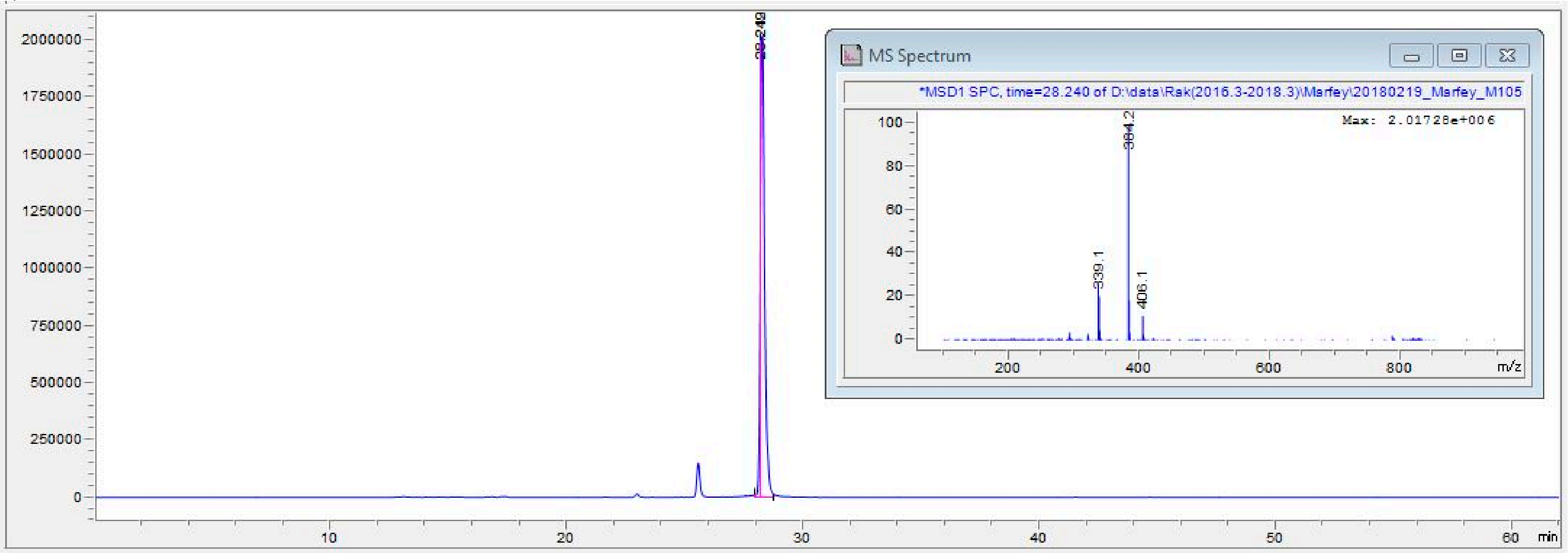

2000000
1750000
1500000
1250000
1000000
750000
500000
250000
0
10
20
30
40
50
60
min
MS Spectrum
*MSD1 SPC, time=28.240 of D:\data\Rak(2016.3-2018.3)\Marfey\20180219_Marfey_M105
Max: 2.01728e+006
100
80
60
40
20
0
384.2
339.1
406.1
200
400
600
800
m/z

Figure S17. Retention times of the L-FDAA derivatized amino acids from compound **1**

1) L-Glu

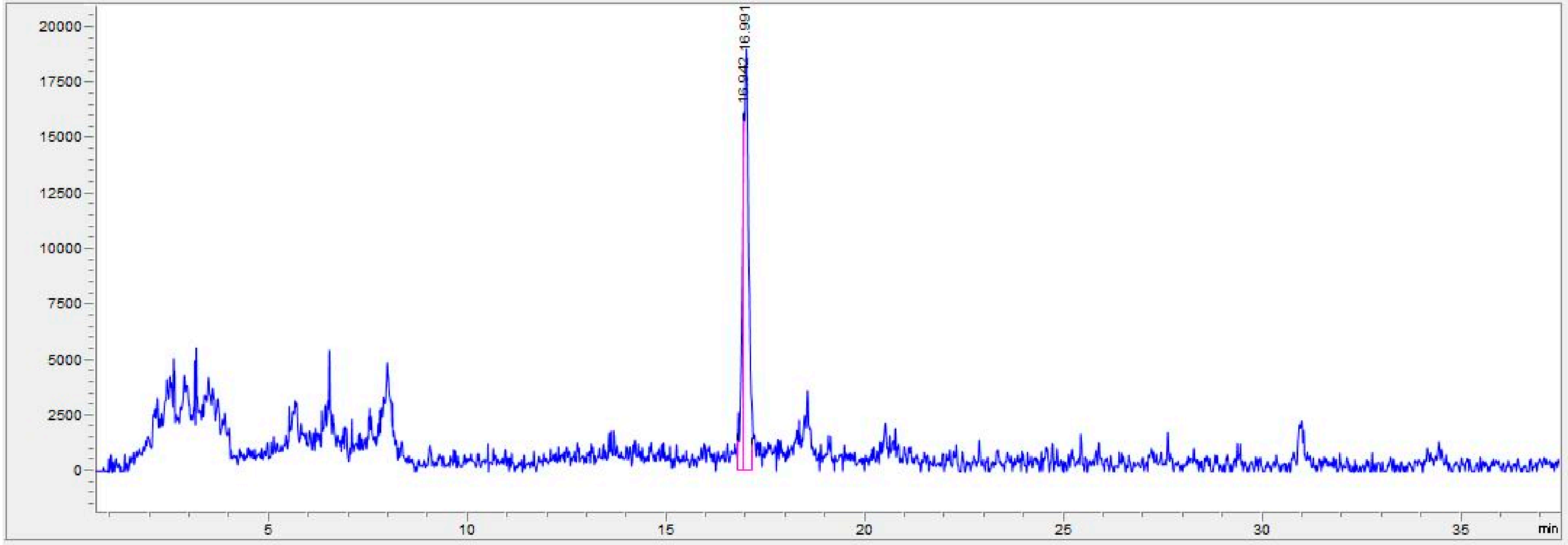

2) L-Phe

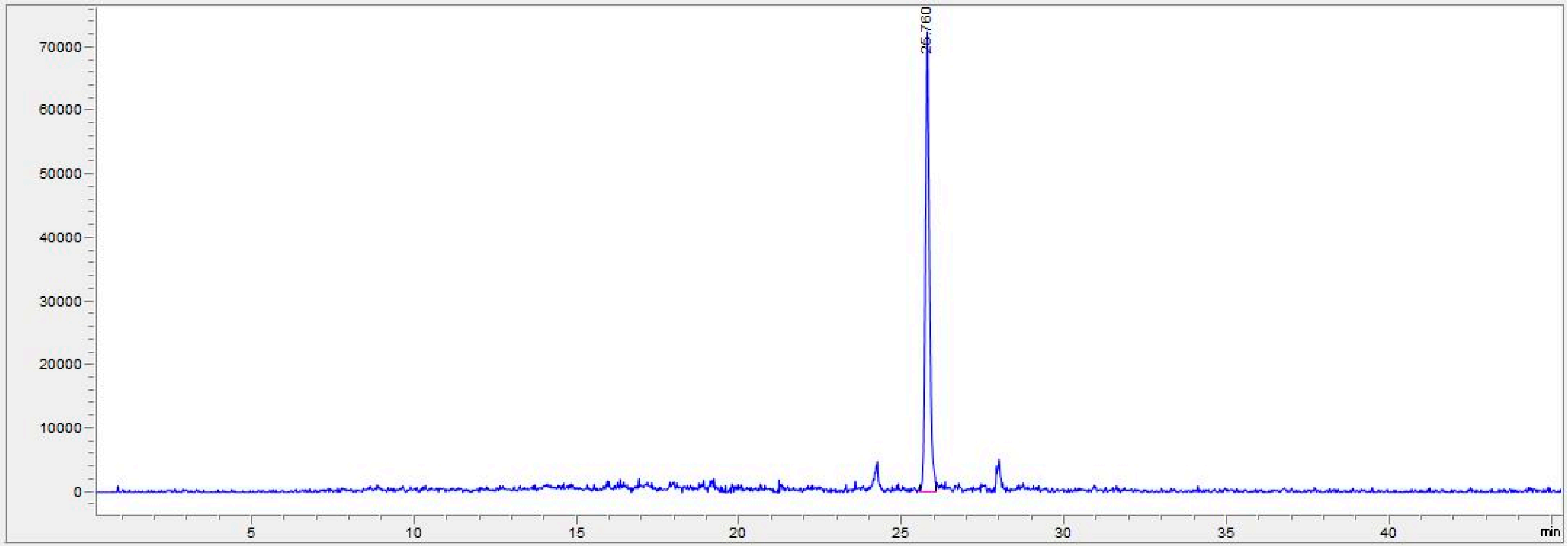

25.760
70000
60000
50000
40000
30000
20000
10000
0
5
10
15
20
25
30
35
40
min

3) L-Val

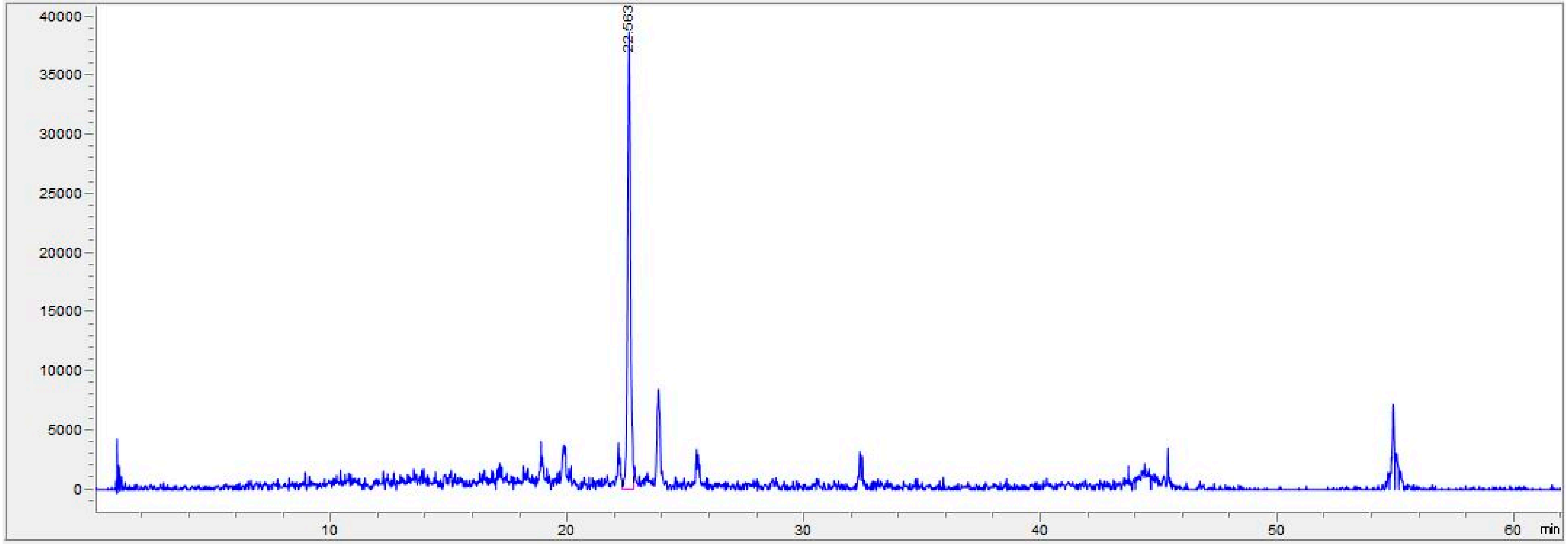

40000
35000
30000
25000
20000
15000
10000
5000
0
22.563
10
20
30
40
50
60
min

Figure S18. Retention times of the L-FDAA derivatized L-Leu from compound **2**

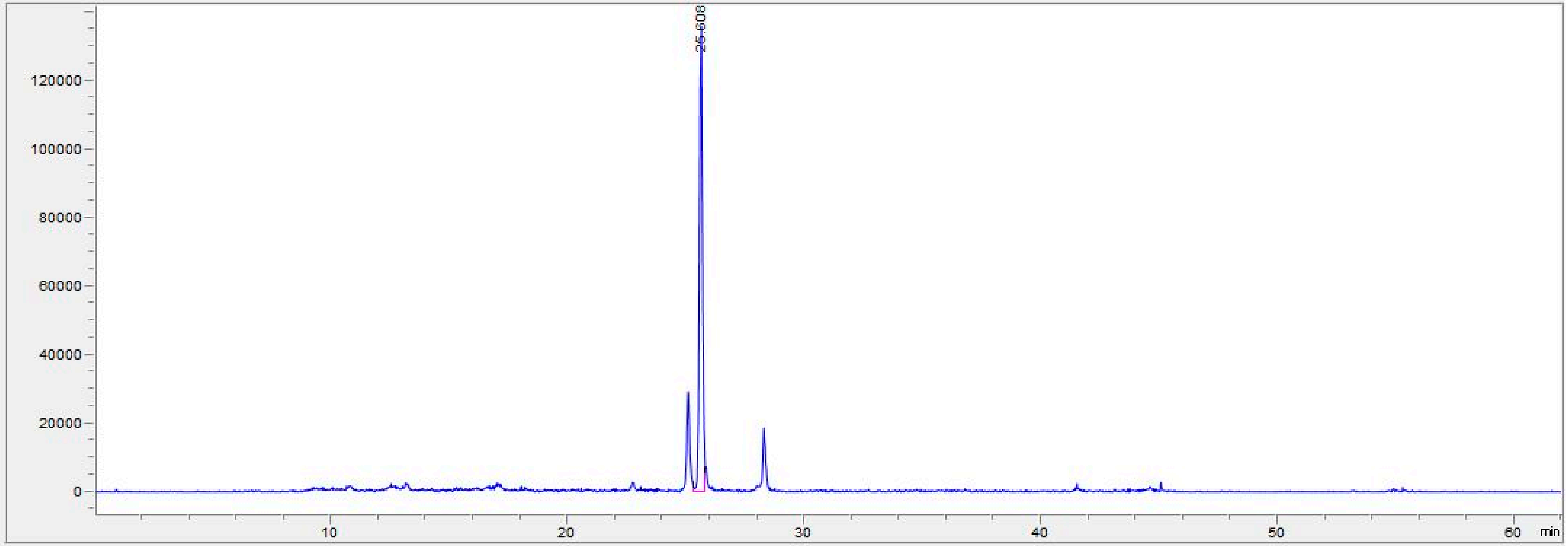

Figure S19. Stimulation of production of compounds **1-3**

Actinomadura sp. RB99 was grown in 500 mL ISP2 broth supplemented with varying NaCl concentrations (1-3%) for 10 days at 30 °C. Culture supernatant was extracted with activated HP20 resin using the following steps: 1. Wash step using 1 L dH_2O, 2. elution with 20% MeOH (500 mL), 50% MeOH (500 mL), 100% MeOH (500 mL) and 100% Acetone (500 mL). Organic solvents were evaporated under reduced pressure and resuspended in 20% MeOH and purified using an activated SPE C18 column using the following step gradient (20% MeOH, 50% MeOH, 80% MeOH, 100% MeOH). SPE fractions were analysed using HRMS and HPLC-UV and main metabolite signals dereplicated using an in-house MS/UV data base.

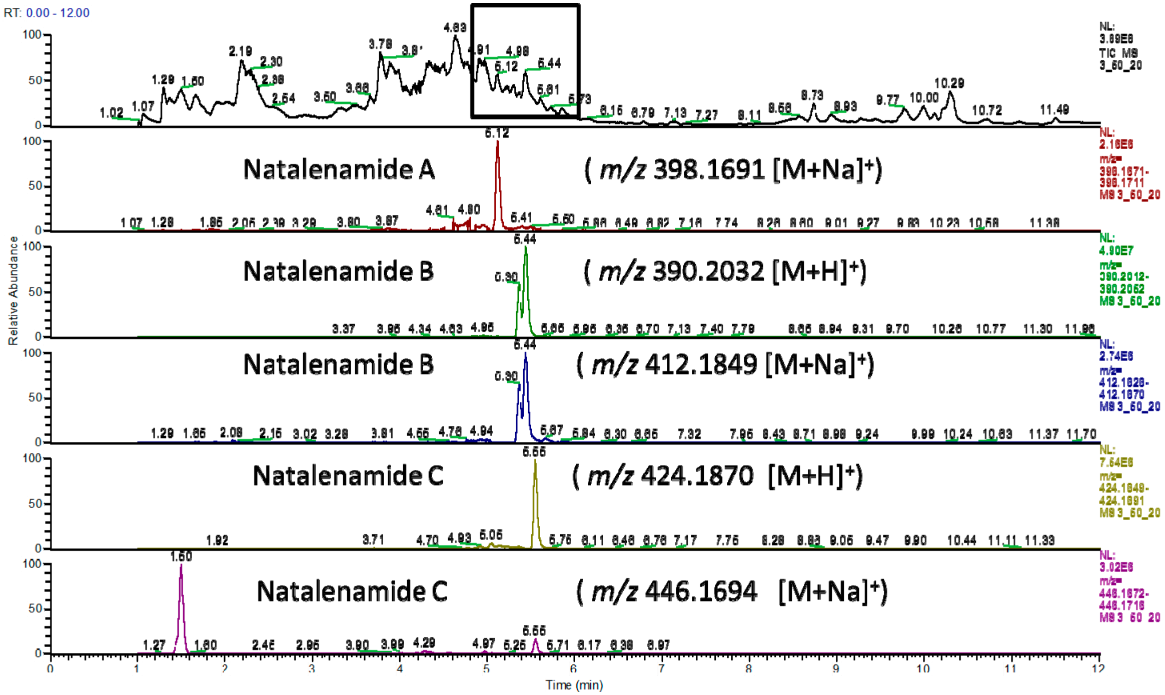

Supplement: Supplementary file 1 [file molecules-23-03003-s001.pdf]
